# Population size as a major determinant of mating system and population genetic differentiation in a narrow endemic chasmophyte

**DOI:** 10.1186/s12870-023-04384-8

**Published:** 2023-08-09

**Authors:** Boštjan Surina, Manica Balant, Peter Glasnović, Ivan Radosavljević, Živa Fišer, Nataša Fujs, Sílvia Castro

**Affiliations:** 1 Natural History Museum Rijeka, Lorenzov prolaz 1, 51000 Rijeka, Croatia; 2 Faculty of Mathematics, Natural Sciences and Information Technologies, University of Primorska, Glagoljaška 8, 6000 Koper, Slovenia; 3 Institut Botànic de Barcelona (IBB, CSIC–Ajuntament de Barcelona), Passeig del Migdia s.n., Parc de Montjuïc, 08038 Barcelona, Spain; 4 Division of Botany, Department of Biology, Faculty of Science, University of Zagreb, Marulićev trg 9a, 10000 Zagreb, Croatia; 5 Centre of Excellence for Biodiversity and Molecular Plant Breeding, Svetošimunska cesta 25, 10000 Zagreb, Croatia; 6 Centre for Functional Ecology–Science for People & the Planet, Department of Life Sciences, University of Coimbra, Calçada Martim de Freitas, 3000-456 Coimbra, Portugal

**Keywords:** Chasmophyte, Conservation genetics, Flower morphology, Hybridization, Introgression, Mating system, Microsatellites, *Moehringia*, Pollination biology, Population size, Selfing syndrome

## Abstract

**Background:**

Mating system is one of the major determinants of intra- and interspecific genetic structure, but may vary within and between plant populations. Our study model included all known populations of *Moehringia tommasinii* (Caryophyllaceae), a narrow endemic plant inhabiting rock crevices in the northwestern Adriatic, and some populations of co-occurring and widespread *M. muscosa*, an ecologically divergent relative with an overlapping flowering period. We performed reciprocal crosses within and between taxa and used molecular markers to assess the extent of gene flow within and between populations and taxa. Using coefficient of inbreeding, population size, seed weight, pollen-to-ovule ratio, and flower display size, we also looked for evidence of a selfing syndrome.

**Results:**

A surprisingly high variation in mating systems was observed among populations of *M. tommasinii*. These populations exhibited genetic structuring, with their size positively correlated with both seed weight and pollen production. Although a selfing syndrome could not be confirmed as the majority of selfing resulted from allogamous treatments, the occurrence of selfing was notable. In the presence of *M. muscosa*, at a site where both species coexist closely, a distinct pattern of fruit production was observed in *M. tommasinii* following various pollination treatments. Molecular and morphometric data provided evidence of hybridization followed by local extinction at this site.

**Conclusions:**

Population size proved to be the most important factor affecting the mating system in genetically structured populations of *M. tommasinii*. Lighter seeds and lower pollen production observed in populations with pronounced selfing do not provide enough evidence for the selfing syndrome. Detected gene flow between *M. tommasinii* and the sympatric *M. muscosa* suggested weak reproductive barriers between the taxa, which could pose a conservation problems for the former species. Hybridization leading to local extinction may also resulted in floral polymorphism and disruption of mating patterns of *M. tommasinii*.

**Supplementary Information:**

The online version contains supplementary material available at 10.1186/s12870-023-04384-8.

## Background

Theoretical and empirical studies agree that mating systems are primary determinants of the extent and organization of intra- and interspecific genetic structure within and among plant populations [1–4]. The loss of heterozygosity upon inbreeding and the differences in the genetic structure and variations between inbred and outbred taxa found ample practical support with the advent of innovative field and laboratory experiments that provided measurements of outcrossing rates, inbreeding coefficients, allelic diversity, and heterozygosity [2]. However, apart from mating systems, patterns of genetic variation within and among plant populations are influenced by evolutionary history [5], demography [6–8], gene flow [9], and life history traits of taxa [10].

Genetic variation within and among populations is also influenced by a range of ecological factors [11, 12], including frequency, behaviour and taxonomic distribution of pollinators [13, 14], which often have confounding effects [2]. As a consequence, considerable genetic variation has been revealed within and among plant species with similar mating systems [11]. In spite of the genetic advantages of outcrossing, the change in mating system from outcrossing to self-fertilization is of great biological importance and is one of the most common evolutionary transitions in angiosperms ([15, 16] and the references therein), although many mechanisms involved in this transition remain poorly understood [17]. In general, selfing is associated with significantly lower effective population size [18], which leads to pollen discounting, inbreeding depression and finally to reduced genetic variation [19] and genetically uniform populations [15]. Consequently, differentiation among selfing populations is expected to be greater than among outcrossing ones [2].

One of the most widely accepted ecological scenarios for the evolutionary transition from outcrossing to selfing is a state of limited pollen/pollinator availability or mate availability, e.g., in case of low plant population size and/or density [6, 20–24]. Alternative views, but not mutually exclusive, hold that selfing results from rapid maturation in marginal habitats [25], polyploidization [26, 27], domestication [28] and reduced spatial and/or temporal separation between flower sexual organs.

The change from allogamy (geitonogamy and xenogamy) to autogamous self-fertilisation is usually associated with a selfing syndrome – a characteristic set of changes in floral morphology and function that promote autonomous selfing [16, 29]. As has been demonstrated in a number of plant genera, predominantly selfing species have less conspicuous floral display in the form of a smaller number and size of flowers and fewer simultaneously open flowers than their outbreeding relatives [5, 30, 31], smaller and lighter seeds [32–35], reduced pollen-to-ovule ratio [36], and lower nectar and scent production [37–40].

Considerable variation in mating systems has been observed among populations, both spatially and temporally [41–43], due to a variety of factors (as mentioned above). Recently, however, researchers have observed that mating systems differ significantly in areas where evolutionarily closely related species co-occur. There, pronounced autonomous selfing was found in populations of one or both sympatric taxa depending on the intensity and symmetry of gene flow [44–46]. Successful transfer of alleles between taxa can contribute to the extinction of one of the taxa through outbreeding depression, demographic swamping and genetic assimilation by an abundant congener [47, 48]. In this scenario, interactions between narrowly distributed endangered taxa and closely related co-occurring taxa become of special concern. A detailed knowledge of the mating systems of endangered plant taxa is essential for successful long-term conservation actions [49]. In outcrossing taxa, genetic diversity is maintained, whereas in predominantly selfing taxa, the fixation of certain alleles can reduce or even halt the response to environmental change; in extreme cases, the loss of genetic diversity can contribute significantly to the extinction of the entire population (e.g. [29, 50–52]).

However, in fragmented and genetically depauperate populations that have evolved differently in contrasting environmental conditions, among-population crosses may result in outbreeding depression by thwarting co-adapted gene complexes [53] or disrupting local adaptations among populations [54, 55].

Assuming that the mating system is a primary, yet not the only, force regulating patterns of genetic variation both within and among populations, and to reduce confounding effects of a variety of ecological, historical, and evolutionary factors, operating within or among populations of phylogenetically closely related taxa [2, 4, 15], we selected all known populations of *Moehringia tommasinii* March., a narrowly distributed chasmophyte inhabiting a preserved, until rather recently human-free, and homogeneous, albeit extreme, habitat as a model system to answer the following questions: (a) What is the extent of variation in mating systems among populations? (b) How do mating systems and population size affect genetic parameters within and among populations? (c) Is there evidence of a selfing syndrome as a result of reduced population size and/or outbreeding depression? Finally, (d) does outcrossing with an evolutionarily closely related but ecologically divergent *M. muscosa* affects the mating system of our model plant?

## Study system

**Fig. 1:**
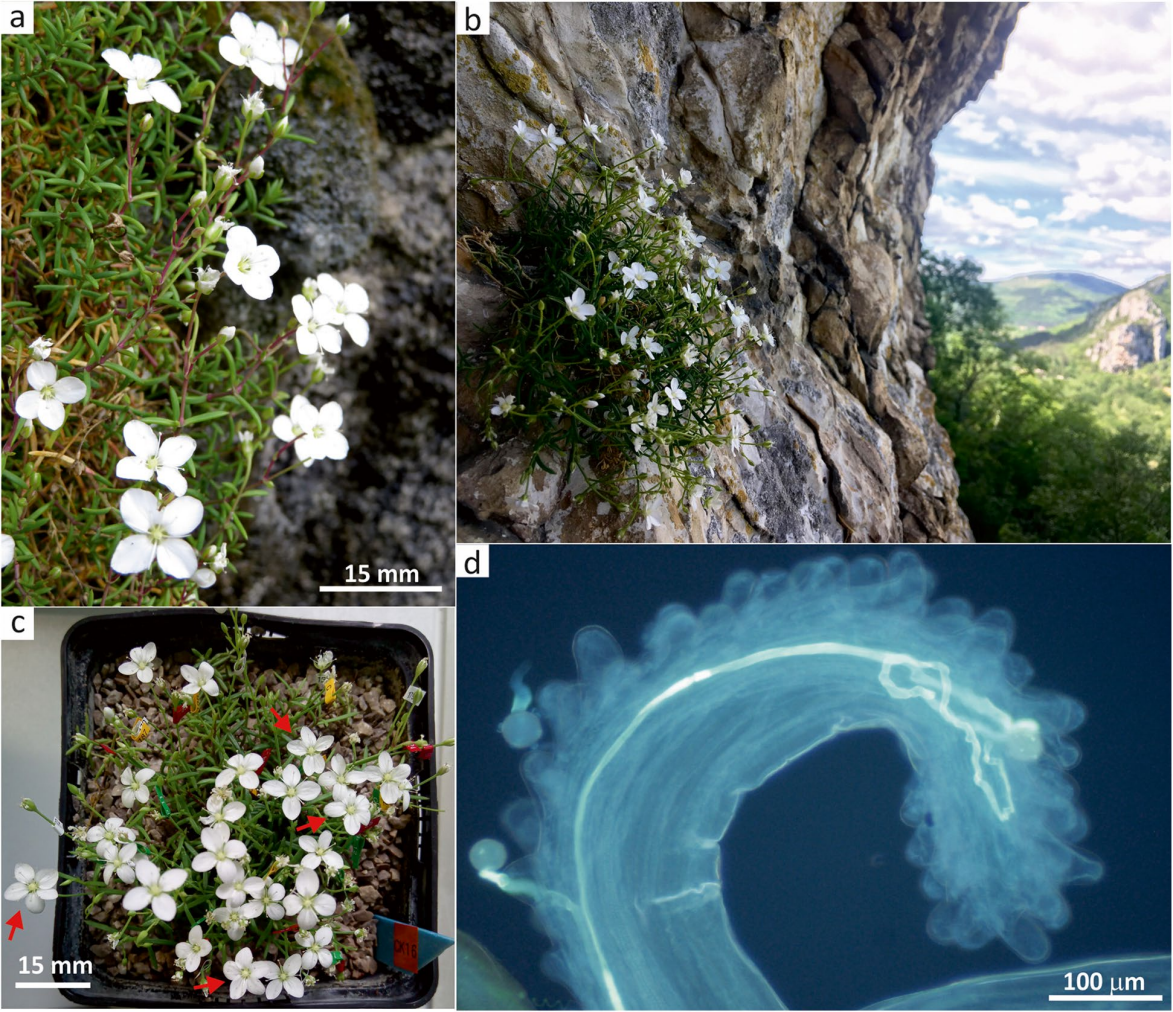
*Moehringia tommasinii* (Caryophyllaceae), a narrow endemic in the north of the Istrian peninsula (NW Adriatic, N Mediterranean). **a** Habitus. **b** Typical growth site – rain-shaded rock crevices in overhanging cliffs. **c** Terratological aberations showing tetramerous and pentamerous flowers (indicated with red arrows) within a single individual. **d** Pollen tube development at the stigmatic papillae and beginning of the style in *M. tommasinii* after hand-pollination experiments. Photo: P. Glasnović – a, Ž. Fišer – b, d, B. Surina – c

Tommasini’s Sandwort – *Moehringia tommasinii* (Caryophyllaceae, Fig. 1a) is a small, caespitose, suffrutescent perennial with short, pendent to procumbent, glabrous, glaucous and somewhat fleshy stems (5–20 cm) occasionally swollen at nodes (fragile when dry). Its leaves (5–20 × 1–1.5 mm) are rather fleshy, linear or elongate-spathulate, keeled and acute. Flowers are white, predominantly tetramerous (Fig. 1c) and arranged in terminal inflorescences as 3–7 flowered cymes. Flower pedicels (8–20 mm) are slightly swollen at apex and become markedly recurved in fruit. Sepals (up to 2.5 mm) are lanceolate with scarious margins, while its white petals (4–7 × 3 mm) are ovate-lanceolate. Subglobose to ovoid capsule is included within calyx. Seeds (1–1.2 mm) with conspicuous and intricately branched strophiole are black and shiny with smooth testa [56].

Judging from morphology and habitat preference, *M. tommasinii* is probably closely related to *M. bavarica* (L.) Gren. (incl. *M. insubrica* Degen), *M. papulosa* Bertol., *M. provincialis* Merxm. & Grau, and *M. dielsiana* Mattf. [57], which was partially confirmed by the results of the molecular phylogeny of the genus, where a basal polytomy of sandworts remained largely unresolved [58]. According to preliminary field observations, the flowers of certain populations of *M. tommasinii*, especially the petal shape, are morphologically similar to the flowers of the sympatric, evolutionary closely related [58] but ecologically divergent *M. muscosa* L., with overlapping flowering periods. Stems of *M. muscosa*, unlike *M. tommasinii*, are green, sometimes shiny when fresh, and its bright green, linear-filiform leaves are never fleshy, while its smaller white petals are linear-lanceolate. However, the morphological characters of chasmophytic sandworts, especially the vegetative ones [57, 59], are affected by a high degree of homoplasy [60] and convergent evolution due to adaptations to specific but very similar environments. *M. tommasinii* and *M. muscosa* both have the same diploid and base chromosome number (2*n* = 24; *x* = 12) [56, 61].

Tommasini’s sandwort is an obligate chasmophyte that prefers crevices of vertical, usually rain-shaded, overhanging limestone cliffs between 60 and 530 m a.s.l. (Fig. 1b). The specific Mediterranean chasmophytic plant community with *M. tommasinii*, referred to as *Asplenio lepidi-Moehringietum tommasinii* Martini 1990, is floristically depauperate and consists of 4–13 plant species with low density and coverage per relevé [62]. According to literature [62–64], *M. tommasinii* occurs in 6 localities in the north of the Istrian peninsula (NW Adriatic, Table 1), covering about 25 km of cliffs in the border area between Italy, Slovenia and Croatia, so that the species extent of occurrence is only 53.1 km^2^. The number of individuals of *M. tommasinii* varies greatly between sites. In all countries the species is red-listed, and the IUCN Red List classifies it as endangered (EN) [65]. The species is listed in Annex II of Council Directive 92/43/EEC on the Conservation of natural habitats and of wild fauna and flora. Unlike *M. tommasinii*, *M. muscosa* is a widespread relative in the mountains of central and southern Europe, preferring shady and somewhat moist rock crevices in forests. It occurs in stands with high coverage of bryophytes within the alliance *Moehringion muscosae* Horvat & Horvatić 1962 [66]. In one locality (GL), individuals of *M. tommasinii* and *M. muscosa* grow only a few meters apart.

**Table 1:** Sampling localities, number of individuals and population genetic parameter estimates for six populations of *Moehringia tommasinii* and three populations of *M. muscosa*

Taxon	Pop.	Lat/Long	Elev. (m)	*N*_ind_	*N*_pg_	*N*_pr_	*N*_ar_	*H*_o_	*H*_e_	*F*_is_
*M. tommasinii*	^1^GL	45^0^37’03.2’’/13^0^52’27.9’’	150	230	30	5	3.31	0.46	0.49	0.08*
	OSP	45^0^34’20.5’’/13^0^51’45.9’’	100	1150	30	7	3.73	0.38	0.41	0.07*
	CK	45^0^32’56.8’’/13^0^53’06.8’’	350	714	30	4	3.33	0.37	0.38	0.02
	PP	45^0^31’43.5’’/13^0^54’11.7’’	325	248	30	12	3.81	0.40	0.44	0.08*
	ISTa	45^0^22’42.2’’/13^0^52’55.4’’	60	2730	30	2	3.15	0.41	0.38	-0.10
	ISTb	45^0^22’43.3’’/13^0^53’41.8’’	140	970	30	4	2.76	0.34	0.36	0.05
*M. muscosa*	^1^GL^m^	45^0^37’03.2’’/13^0^52’27.9’’	150	/	16	5	3.17	0.41	0.55	0.24*
	VDC^m^	45^0^28’26.4’’/14^0^04’59.3’’	780	/	21	3	3.22	0.48	0.51	0.05*
	OBR^m^	45^0^28’13.6’’/14^0^27’23.4’’	1170	/	/	/	/	/	/	/

*Pop*.–population’s acronyms, Lat/Long in *WGS84*, Elev.–elevation, *N*_ind_–number of individuals per population, *N*_pg_–number of samples for population genetic analysis per population, *N*_pr_–number of private allels, *N*_ar_–allelic richness, *H*_o_–detected heterozygosity, *H*_e_–expected heterozygosity, *F*_is_–within-population fixation index, ^1^Sympatric population with *M. tommasinii* and *M. muscosa*, * significance at the *p* < 0.05, dash indicates lack of information

## Results

### Flower life-span and sexual functioning

**Fig. 2:**
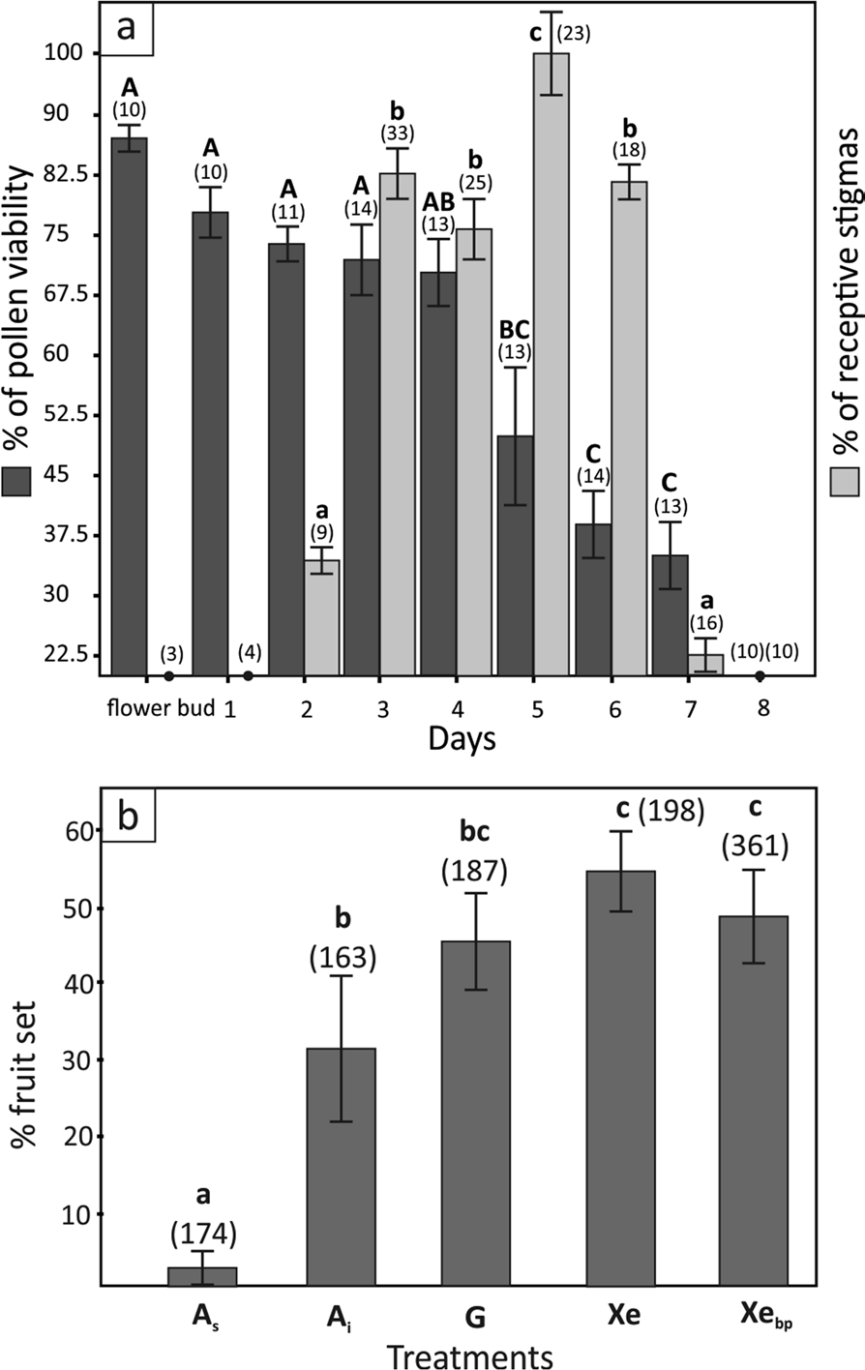
Flower biology and mating system of *Moehringia tommasinii*. **a** Sexual functioning of flowers of *Moehringia tommasinii*. Pollen viability (dark grey bars) is given as the mean percentage (± SE) of stained pollen grains and stigma receptivity (light grey bars) as mean percentage (± SE) of receptive stigmas with germinating pollen grains, both as a function of flower age. Different letters indicate statistically significant differences in male (upper case) and female (lower case) function according to flower age (*p* < 0.05). Numbers in parentheses indicate the number of flowers manipulated. **b** Controlled hand-pollination experiments conducted in *M. tommasinii* to examine the mating system drawn across all six populations. Fruit set is given as the mean percentage (± SE) of flowers that set fruit after a given pollination treatment. Pollination treatments: A_s_–spontaneous selfing, A_i_–induced selfing, G–geitonogamy, Xe–xenogamy, Xe_bp_–between population crosses. Different letters indicate statistically significant differences in male (upper case) and female (lower case) function according to flower age (*p* < 0.05). Numbers in parentheses indicate the number of flowers manipulated

The flowers of *M. tommasinii* generally lasted for 7 days (Fig. 2a). The anthers dehisced in day 1 and pollen was available in the anthers until the flowers withered. However, the highest pollen germination was observed in the buds one day before flowers opened and decreased steadily until the middle of anthesis and significantly after day 5 (χ^2^ = 21.91, *p* < 0.001). From the observation of the flowers of the potted plants in the garden, pollen was almost completely washed from the opened anthers when they were directly exposed to rain. Stigmas became receptive on day 2 and receptivity increased significantly on days 3–6 (χ^2^ = 48.78, *p* = 0.001324) with the highest stigmatic receptivity being observed on day 5, when stigmatic lobes, which now had distinct papillae on receptive surface, were fully opened (Fig. 1d). The stigma receptivity decreased significantly on day 6. Overlap of male and female functions was observed on days 2–7, while high levels of pollen germinability and stigma receptivity coincided on days 3–5.

### Population sizes, mating systems and pollen limitation

Population size varied from 230 (population GL, followed by population PP with 248) to 2730 individuals (population ISTa; Table 1).

**Fig. 3:**
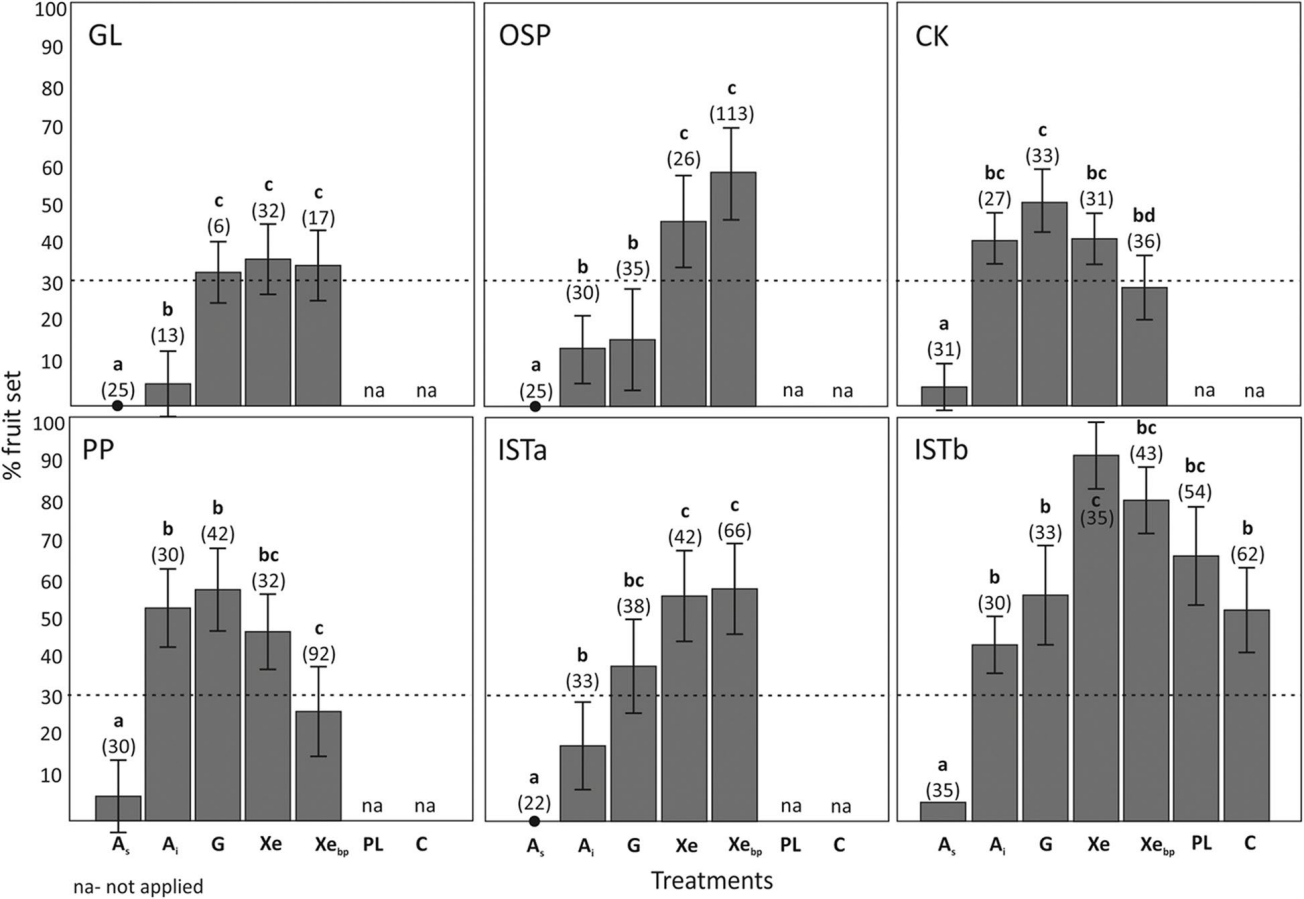
Controlled hand-pollination experiments conducted in *Moehringia tommasinii* to study the mating system separately for the populations GL, OSP, CK, PP, ISTa and ISTb and to assess pollen limitation (ISTb only). Fruit set is given as the mean percentage (± SE) of flowers that set fruit after a given pollination treatment. Pollination treatments: A_s_–spontaneous selfing, A_i_–induced selfing, G–geitonogamy, Xe–xenogamy, Xe_bp_–between population crosses, PL–pollen limitation, C–control, na–not applied. Different letters indicate statistically significant differences in male (upper case) and female (lower case) function according to flower age (*p* < 0.05). Dashed line indicates fruit set obtained by crossings between *M. tommasinii* and *M. muscosa*. Numbers in parentheses indicate the number of flowers manipulated

**Fig. 4:**
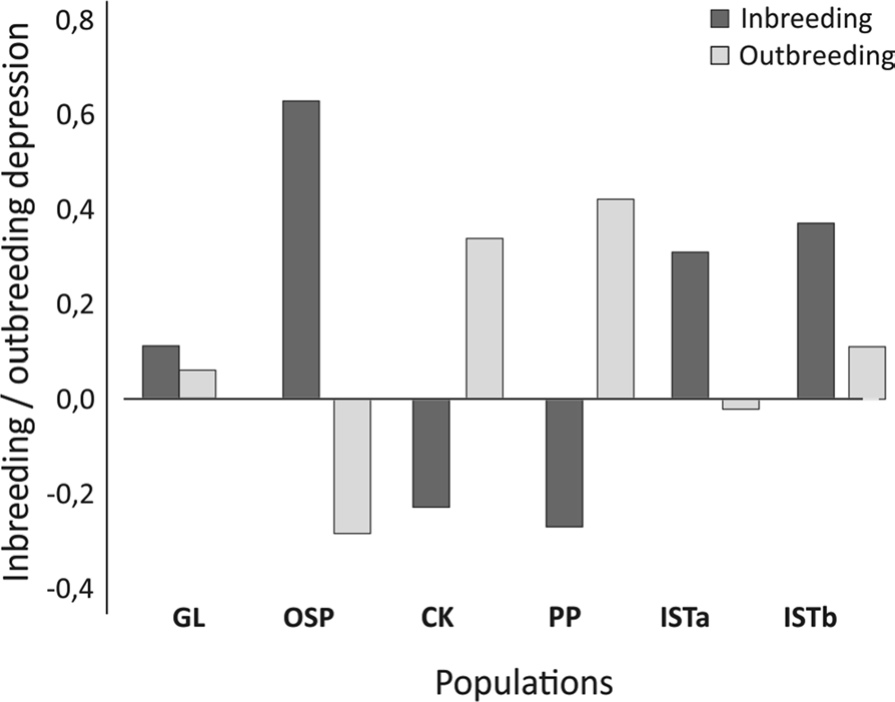
Coefficients of inbreeding depression (after [67]) and outbreeding depression (after [68]) in *Moehringia tommasinii* for populations GL, OSP, PP, ISTa, and ISTb. Higher positive values of inbreeding and outbreeding coefficients indicate higher preferences to outcrossing and selfing, respectively

Pollination treatments significantly affected fruit set in *M. tommasinii* across populations (χ^2^ = 76.75, *p* < 0.001, Fig. 2b, Additional file 1: Table S1). Spontaneous autogamy (A_s_) resulted in significantly fewer fruits than the other treatments (*p* < 0.05), while induced autogamy (A_i_) produced significantly fewer fruits than crosses between individuals within (Xe) and between populations (Xe_bp_) (*p* < 0.05). Geitonogamous crosses (G) resulted in neither significantly higher fruit set compared to induced autogamy nor significantly lower fruit set compared to xenogamy (*p* > 0.05). However, substantial variations in mating systems were observed within and between populations (Figs. 3 and 4, Additional file 2: Table S2, Additional file 3: Table S3). Overall, fruit set was higher in allogamous treatments compared to induced autogamous treatments in populations OSP, ISTa and ISTb. In contrast, induced autogamous (A_i_) and/or geitonogamous treatments (G) resulted in higher fruit set than xenogamous treatments between populations (Xe_bp_) in populations PP (treatments A_i_ and G vs. Xe_bp_) and CK (treatments G vs. Xe_bp_).

Fruit set in pollen supplemented flowers (PL) in population ISTb was significantly higher then spontaneous autogamy (A_s_) and, although being lower than outcrossed treatments, no significant differences were detected when comparing with the remaining treatments, including the open-pollinated treatment (C; treatments PL and C applied only in population ISTb). With the exception of xenogamously (Xe) pollinated flowers (χ^2^ = 2.2718, *p* = 0.008), fruit set in open-pollinated flowers (C) was not significantly different from that obtained by hand crossings.

Crosses between *M. tommasinii* and *M. muscosa* resulted in 30% fruit set (indicated by the dashed line in Fig. 3, Additional file 3: Table S3), which was significantly higher than fruit set in spontaneously self-pollinated flowers (A_s_) in all populations and higher than in induced self-pollinated flowers (A_i_) in populations GL, OSP, ISTa, geitonogamously pollinated flowers (G) in population OSP, and between population crosses (Xe_bp_) in populations CK and PP of *M. tommasinii*. On the other hand, significantly higher fruit set was obtained in *M. tommasinii* compared to interspecific crosses in geitonogamous (PP and ISTb) and xenogamous treatments (ISTa&b–Xe and OSP–Xe_bp_) and in pollen-supplemented flowers (ISTb). Fruit set was also higher in open-pollinated flowers (C; treatment applied only in population ISTb) then in interspecific crosses (Xe_bp_), but the difference was not statistically significant (χ^2^ = 2.971, *p* = 0.085).

Xenogamous treatments resulted in higher fruit set compared to autogamous treatments in populations OPS, ISTa, and ISTb (Fig. 4). However, it is noteworthy that populations CK and PP exhibited lower fruit set under both treatment conditions. The highest values of the inbreeding depression coefficient (*δ*_*i*_) were observed in the population OSP (0.629), followed by populations ISTa and ISTb (0.309 and 0.370, respectively), while the lowest values were observed in the populations PP (-0.270) and CK (-0.228). In contrast, the highest values of the outbreeding depression coefficient (*δ*_*o*_) were observed in the populations PP (0.420) and CK (0.338), while the lowest were observed in the populations OSP (-0.285) and ISTa (-0.021).

### Gene flow and population genetic structuring

Population-genetic analysis revealed the absence of substantial differences in levels of observed (*H*_o_) and expected heterozygosity (*H*_e_) among studied *M. tommasinii* populations (Table 1). The lowest values were detected in the ISTb population (0.34 and 0.36, respectively), while the highest values were revealed in the population GL (0.46 and 0.50, respectively). Similar levels of observed and expected heterozygosity were detected in both *M. muscosa* populations, as well. Within-population fixation index (*F*_is_) had positive and significant values in three *M. tommasinii* populations (OSP, PP, and GL), and both *M. muscosa* populations. *F*_is_ showed no correlation with floral display size (Fig. 7a), but appeared to be negatively correlated with population size (Fig. 7d, *p* < 0.001).

**Fig. 5:**
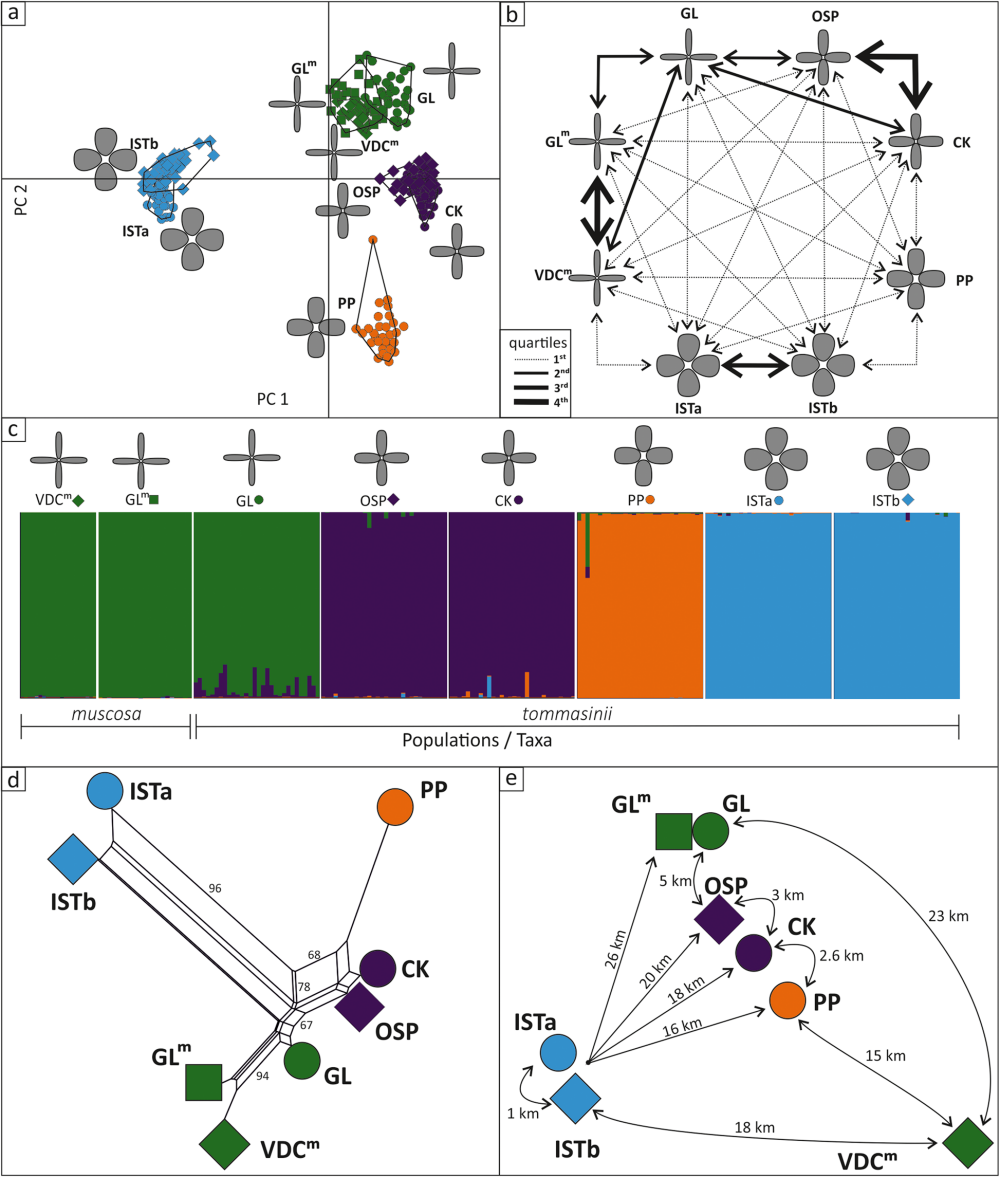
Population genetics of *Moehringia tommasinii*. **a** Principal component analysis of individuals of *Moehringia tommasinii* (populations GL, OSP, CK, PP, ISTa and ISTb) and *M. muscosa* (populations GL^m^ and VDC^m^) based on microsatellite data. **b** Pairwise gene flow between populations of *M. tommasinii* and *M. muscosa*; the lines indicate intensity of gene flow (*N*_m_): 1st quartile – 0.33–0.79, 2nd quartile – 0.80–1.25, 3rd quartile – 1.26–1.71, 4th quartile – 1.72–2.2. **c** Genetic structure and assignment of individuals into classes as assessed by the computer programs STRUCTURE. Each individual plant is represented by a single vertical line; each colour represents a cluster, and the length of the coloured segments indicates the individual’s estimated proportions of membership in those clusters. **d** Neighbour-joining network based on Nei’s genetic distance data-matrix. **e** Simplified geographic position and distances among the populations. Symbols and colours in **a**–**d** designate genetic clusters as inffered from Bayesian assignment test (**c**). In  **a**–**c** petal shape and size are shematically presented for each population of *M. tommasinii* and *M. muscosa*

Allelic richness (*N*_ar_) levels were almost equally distributed among populations, ranging from 2.75 in population ISTb to 3.80 in population PP. Finally, in all populations private alleles (*N*_pr_) were detected, ranging from only two in population ISTa to 12 in population PP. For tests of isolation by distance among populations, Pearson method (*p* = 0.0084, std. obs.: 3.49, expectation: 0.002, variance: 0.035) yielded significant results. Populations ISTa and ISTb on one side, and PP on another, were strongly differentiated when compared to the remaining populations (Fig. 5acd). Populations VDC^m^ and GL^m^ of *M. muscosa* differed to a lesser extent, which was surprising given that they are representatives of other species and the most spatially distant populations in the analyses (Fig. 5e). Population GL of *M. tommasinii* was closely positioned to populations belonging to *M. muscosa* (populations VDC^m^ and GL^m^), while populations CK and OSP of *M. tommasinii* seemingly formed a distinct group. Gene flow (*N*_m_) among populations of *M. tommasinii* ranged from 0.33 (populations PP–ISTb) to 2.17 (OSP–CK; Me = 0.55; Fig. 5b, Additional file 4: Table S4). Relatively high gene flow (> 75% quartile *N*_m_) was observed between populations OSP–CK and VDC^m^–GL^m^ (1.78) for *M. tommasinii* and *M. muscosa*, respectively, and high (75% > quartile *N*_m_ > 50%) was observed between populations ISTa–ISTb (1.70) of *M. tommasinii*. Moderate gene flow (25% < quartile *N*_m_ < 50%) was observed between populations GL–OSP (1.21) and GL–CK (0.99) within *M. tommasinii* and populations GL–GL^m^ (1.23) and GL–VDC^m^ (1.24) between *M. tommasinii* and *M. muscosa*. Low gene flow (< 25% quartile *N*_m_) was observed in all other population pairs within and between species. In general, gene flow between two, albeit distant populations of *M. muscosa* (1.78) was higher than between populations of *M. tommasinii* (median = 0.47, min = 0.34: PP–ISTb, max = 2.17: OSP–CK), while median values of a gene flow between *M. muscosa* and *M. tommasinii* were 0.55 and 0.58, respectively, when calculated for the populations VDC^m^ and GL^m^. The PCA (Fig. 5a), detected gene flow (Fig. 5b) and especially the results of Bayesian assignment test (Fig. 5c) supported such observations. The neighbour-joining network based on Nei’s genetic distances (Fig. 5d, Additional file 5: Table S5) demonstrated a strong genetic differentiation among the populations. The most probable *K* value in the Bayesian assignment test was four, representing three *M. tommasinii* and additional one *M. muscosa* genetic cluster. The first one comprised of populations ISTa and ISTb, the second of populations CK and OSP, the third of population PP, while the last one included populations of *M. muscosa* (localities VDC^m^ and GL^m^) together with population GL of *M. tommasinii*. The population GL was characterized by a somewhat admixed structure with a strong predominance of *M. muscosa* over the CK–OSP genetic cluster. Such a result indicates not only the presence of interspecific hybridization between the two closely related species, but also the almost complete introgression of the smaller population of *M. tommasinii* (GL) by the more abundant and widespread one of *M. muscosa*. With exception of the population GL, the remaining populations of *M. tommasinii* were characterized by very low admixture levels, thus suggesting the prolonged isolation and the absence of any substantial gene flow among populations.

### Floral display

**Fig. 6:**
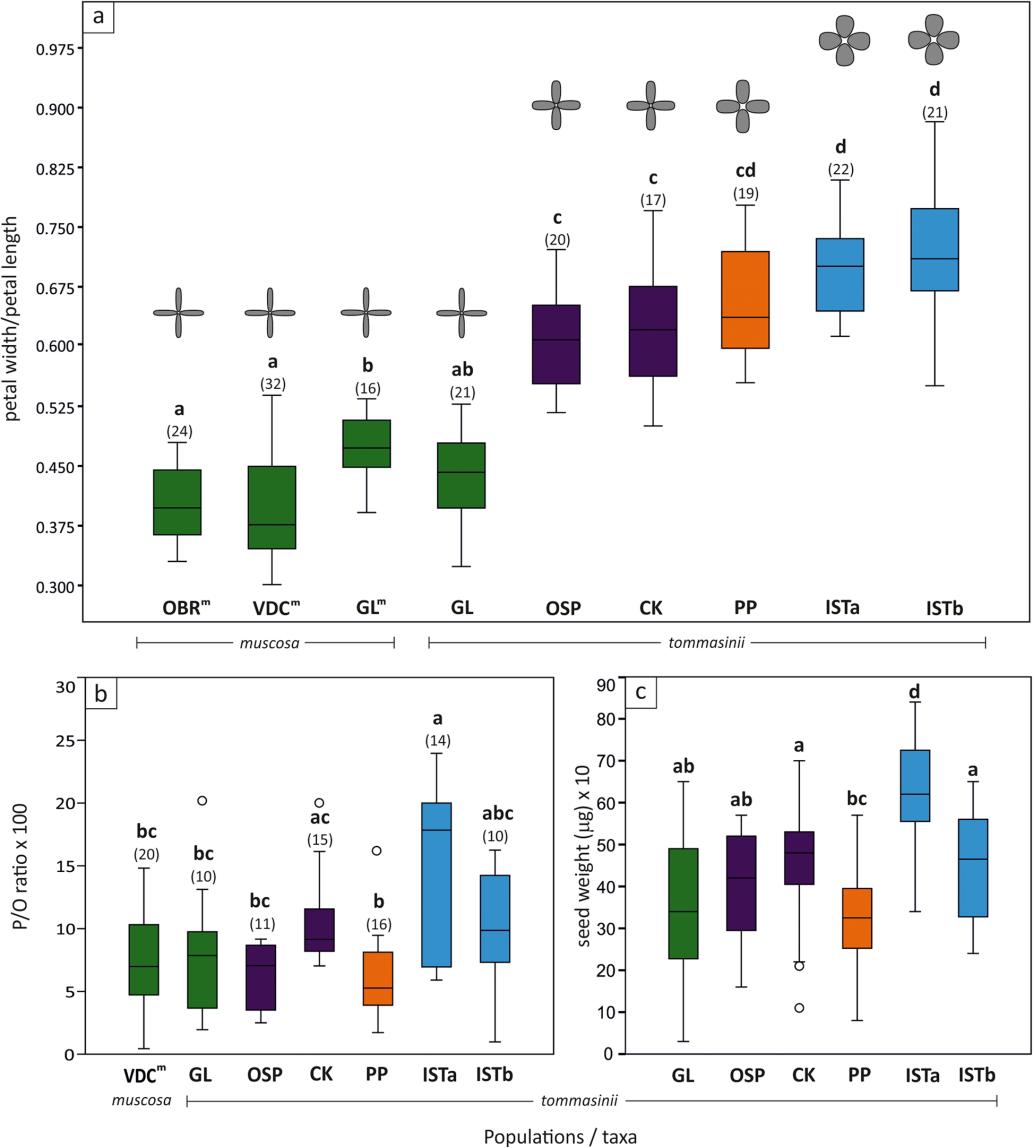
Characteristics of floral display, pollen-to-ovule (P/O) ratio and seed weight of populations of *Moehringia tommasinii* and *M. muscosa.* **a** Petal index (petal width / petal length) of *M. muscosa* (populations OBR^m^, VDC^m^ and GL^m^) and *M. tommasinii* (populations GL, OSP, CK, PP, ISTa and ISTb). **b** P/O ratio of *M. muscosa* (population VDC^m^) and *M. tommasinii* (populations GL, OSP, CK, PP, ISTa and ISTb). **c** Seed weight (µg) of *M. tommasinii* for populations GL, OSP, CK, PP, ISTa and ISTb. Box colours are in agreement with the results of genetic clustering of individuals as in Fig. 5. Length of whiskers indicate standard error (one sigma). In Fig. 5a petal shape and size are shematically presented for each population of *M. tommasinii* and *M. muscosa*

Petal size and shape differed significantly between populations of *M. tommasinii* and *M. muscosa* (χ^2^ = 153, *p* < 0.0001, Additional file 5: Table S5, Additional file 6: Table S6). Petal index (petal width / petal length) varied from 0.31 to 0.98 in *M. muscosa* (population OBR^m^) and *M. tommasinii* (population ISTb), respectively (Fig. 6a). The largest and most oval to obovate petals were observed in populations ISTa and ISTb, followed by population PP. Significantly smaller flowers with ovate-lanceolate petals were observed in the second group formed by populations OSP and CK. The smallest petal indices with lanceolate petals were observed in three populations of *M. muscosa* (OBR^m^, VDC^m^, and GL^m^) and one population of *M. tommasinii* (GL). In general, *M. muscosa* had significantly smaller petals as well as petal index compared to mean population values of *M. tommasinii*.

### Pollen-to-ovule ratio

**Fig. 7:**
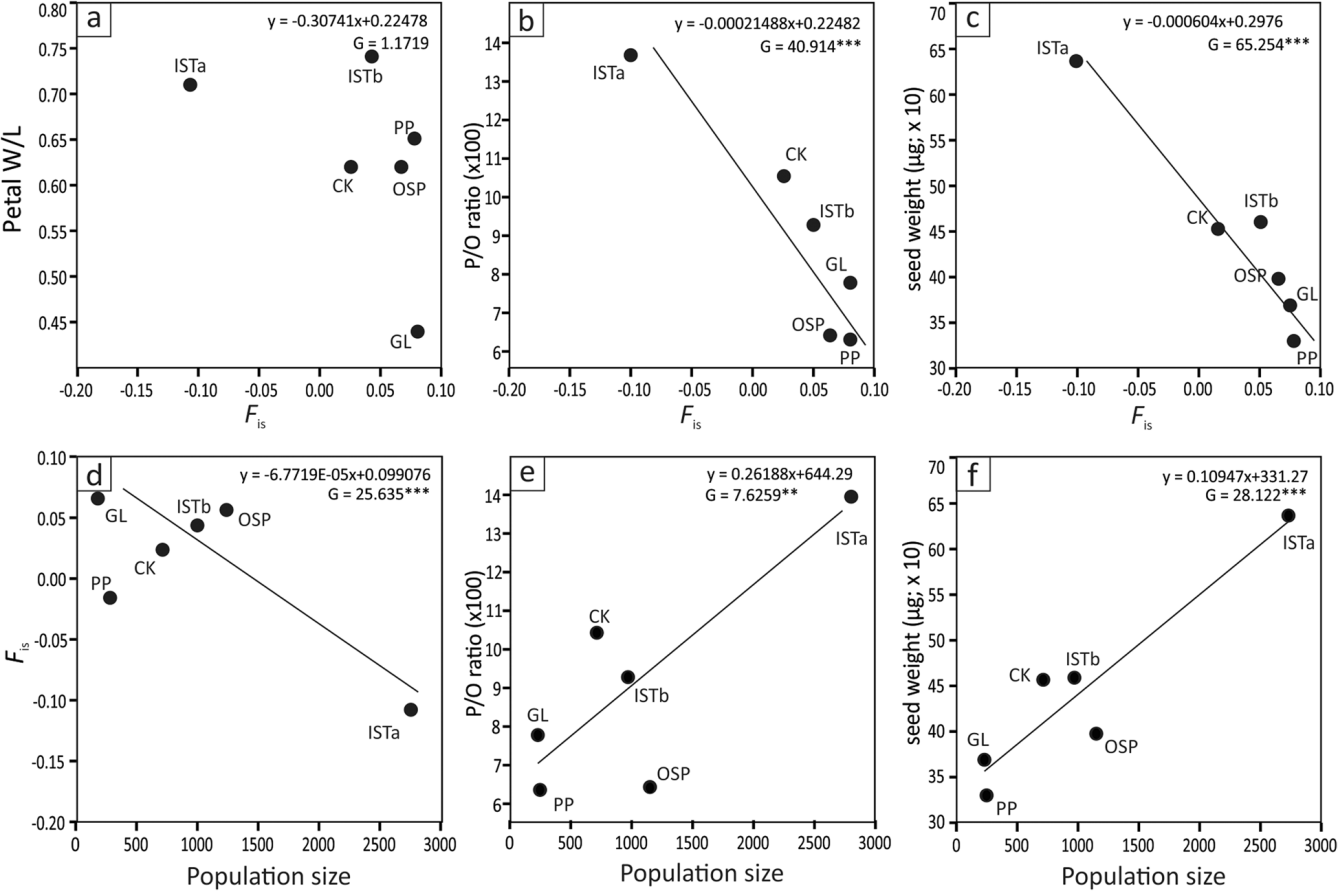
Generalized linear models using normal distribution and identity link of within-population fixation index (*F*_is_: **a–c**) and population size (**d–f**) according to petal index (Petal W/L: **a**), within-population fixation index (*F*_is_: **d**), pollen-to-ovule ratio (P/O ratio: **b, e**), and seed weight (µg: **c, f**) for all populations of *Moehringia tommasinii*. * *p* < 0.05, ** *p* < 0.01, *** *p *< 0.0001

Pollen-to-ovule ratios varied widely within and among populations and taxa (χ^2^ = 25.8, *p* < 0.005, Table 2; Fig. 6b, Additional file 7: Table S7). The lowest P/O ratio was obtained for the population of *M. muscosa* from locality VDC^m^ (43.8), and the highest for *M. tommasinii* from locality ISTa (2395.8). For *M. muscosa*, the P/O ratio varied from 43.8 to 1482.1 (mean = 743.0, while for *M. tommasinii* it ranged from 98.2 (population ISTb) to 2395.8 (population ISTa). The highest values and variation in P/O ratios were observed in population ISTa (590.3–2395.8, mean = 1522.0), which were significantly higher than values obtained in populations OSP, PP, GL for *M. tommasinii* and VDC^m^ for *M. muscosa*. Although P/O values were different in other populations, the differences were not statistically significant. The highest variation in the number of stamens was observed in the populations GL (4–10 stamens; coeff. var. 23.24%), and no teratological aberrations were observed in the populations OSP and ISTb (8), whereas in the populations CK (8–9), PP (6–9), and ISTa (7–11) the variation was low to moderate, reaching 4.3%, 7.2%, and 11.7%, respectively (Table 2). Consequently, P/O ratio in *M. tommasinii* was the most variable in population GL (66.8%), while the least variable was in population CK (30.6%). P/O ratio was significantly negatively correlated with within-population fixation index (Fig. 7b, *p* < 0.001). In contrast, P/O ratio was significantly positively correlated with population size (Fig. 7e, *p* < 0.01).

**Table 2:** Sampling localities and number of stamens, ovules and pollen grains per individual flowers of *Moehringia tommasinii* and *M. muscosa*

Taxon	Pop.	N_fl/pop_	N_st/fl_	N_ov/fl_	N_p/fl_	P/O ratio	Log P/O ratio
*tommasinii*	GL	10	(4) **7.3** (10)	(3) **6.6** (8)	(1562) **4500** (10,500)	(195.3) **777.5** (1895.8)	(2.3) **2.8** (3.3)
	OSP	11	(8) **8** (8)	(6) **7.5** (8)	(1750) **4767.1** (7125)	(250) **641** (916.7)	(2.4) **2.8** (3.9)
	CK	15	(8) **8.1** (9)	(5) **7.3** (8)	(4188) **7491.7** (10,625)	(703.1) **1043** (1800)	(2.9) **3** (3.3)
	PP	16	(6) **7.9** (9)	(5) **7.5** (8)	(1375) **4578.1** (8000)	(171.9) **635.5** (1600)	(2.2) **2.8** (3.2)
	ISTa	15	(7) **8.4** (11)	(5) **8.1** (13)	(125) **11087.5** (19,625)	(25) **1422.2** (2395.8)	(1.4) **3** (3.4)
	ISTb	11	(8) **8** (8)	(7) **7.6** (9)	(188) **6965.9** (12,813)	(26.8) **896.2** (1625)	(1.4) **2.8** (3.2)
*muscosa*	VDC^m^	20	(7) **8.1** (10)	(6) **8.3** (12)	(438) **5928.1** (10,375)	(43.8) **743** (1482.1)	(1.64) **2.8** (3.2)

N_fl/pop_–number of examined flowers per population, N_st/fl_–number of stamens per flower, N_ov/fl_–number of ovules per flower, N_p/fl_–number of pollen grains per flower, (min) mean (max)

### Seed weight

Seed weight between populations differed significantly (χ^2^ = 59.46, *p* < 0.0001). The lightest seeds were observed in the population PP (mean = 369 µg), and the heaviest in the population ISTa (mean = 627 µg), which also differed significantly from all other populations of *M. tommasinii* (Fig. 6c). Seed weight was negatively related to within-population fixation index (*F*_is_; *p* < 0.001, Fig. 7c) and positively related to population size (*p* < 0.001, Fig. 7f).

## Discussion

### Flower biology and mating system

Mating systems in genus *Moehringia* are very variable. Judging by pollen-to-ovule ratios, *M. pentandra* is cleistogamous to obligately autogamous, whereas *M. trinervia, M. minutiflora*, and *M. glaucovirens* are obligately to facultatively autogamous [69]. In addition, Mayer [70] and Scheffknecht et al. [71] found through controlled hand pollination that an animal vector is required for successful self-pollination in *M. trinervia* and *M. ciliata*, while Hind [69] found that *M. muscosa, M. ciliata, M. tommasinii, M. intermedia, M. markgrafii, M. insubrica*, and *M. sedoides* are facultatively autogamous to facultatively xenogamous. However, our values for the P/O ratio of *M. muscosa* and *M. tommasinii* differ substantially from those reported by Hind [69], and suggest that both species are facultatively xenogamous to xenogamous. This was also largely confirmed by our controlled hand pollinations and by pollen viability and stigma receptivity. However, recent research has shed light on the impact of pollinator dependence and pollination efficiency on the evolution of pollen number per flower, indicating that their influence is more pronounced in this aspect compared to ovule number [72]. Interestingly, in the case of *M. tommasinii*, the number of pollen grains per flower aligns with the observed pollen-ovule ratios.

The flowers of *M. tommasinii* are protandrous, although a clear overlap of sexual phases was observed between days 3 and 4. Consequently, the differences in fruit set between autogamous and geitonogamous treatments observed in all populations, although not statistically significant, could be due to functional temporal separation between the male and female phases. Additionally, due to spatial separation of sexual organs, the species has a low capacity for spontaneous self-pollination (A_s_) and requires pollen vectors for successful pollination. In general, fruit set under cross-pollination (Xe, Xe_bp_) was not significantly higher than under geitonogamy (G) and induced autogamy (A_i_), suggesting that there is the lack of strong self-incompatibility in *M. tommasinii*.

### Inbreeding and outbreeding depression

Results from autogamous treatments pointed to outbreeding depression in the populations CK and PP. Outbreeding depression is usually interpreted as the result of either the breaking up co-adapted gene complexes [53] or the disruption of local adaptation, which is more common in large populations with more than 1000 flowering individuals [54]. The extent of outbreeding depression is influenced by intrinsic and extrinsic factors, e.g. the ecological, physiological, and genetic history of populations, including the extent of gene flow, inbreeding, genetic drift, and selection [73]. Contrary to expectations, *M. tommasinii* in populations CK and PP showed a pronounced degree of outbreeding depression and relatively higher success of autogamous treatments. Inbreeding depression was observed in populations OSP and ISTa with more than 1000 flowering individuals. Here, allogamous treatments resulted in significantly higher fruit set. At the same time, no significant differences in community assemblages and site parameters were observed, indicating specific yet uniform ecological conditions among populations (cf. [62]).

The high variation in outbreeding and inbreeding depression in *M. tommasinii* might be better explained by population sizes and very limited extant gene flow among spatially isolated and therefore genetically differentiated populations (see [74–76]). Pollen flow among populations would increase genetic exchange and reduce population genetic structuring and inbreeding depression [77]. Consequently, the progeny would experience significant advantages through crossing between populations, as deleterious alleles that are otherwise fixed in parental populations remain concealed within hybrid populations as heterozygotes, resulting in the phenomenon of heterosis [78].

Indeed, fruit set in predominantly outcrossing and large populations (OSP, ISTa, ISTb) of *M. tommasinii* resulted in higher seed weights compared to smaller populations (CK, PP) that exhibited higher outbreeding depression, as observed, for example, in *Crinum erubescens* [32], *Hydrophyllum appendiculatum* [33] and *Primula* spp. [79]. Although outbreeding depression usually occurs as a result of crosses between widely separated populations (e.g. [75]), in *M. tommasinii* it occurred at scales of approximately 1−5 km, although even smaller scales have been reported for other plant taxa (e.g. [73, 76, 80, 81]). However, the extent of inbreeding and outbreeding depression based on within-population fixation index and mating system, was highly correlated with the size of individual populations, again confirming the fundamental importance of population size for evolutionary theory [54, 82].

Although analyses were performed on all extant populations of *M. tommasinii*, the limited number of populations available for analysis means that results should be interpreted with some caution. In addition to population size, plant density has been repeatedly recognised as one of the most important factors affecting genetic structure and the extent of inbreeding and outbreeding within and between populations [83, 84], and significantly influencing pollinator foraging behaviour [6, 85–88]. Similar to low population size, low population density can lead to pollen and/or pollinator limitation and selection for floral traits and plant mating system assuring sexual reproduction under these conditions [86, 89, 90]. Higher plant density may result in attracting more pollinators (e.g. [91]), while lower density may result in their dilution [92]. Unfortunately, due to the general inaccessibility of plants and sites, we were unable to assess the effects of plant density and pollinator behaviour on the mating system and genetic structuring of populations of *M. tommasinii.* Pollen supplementation to open flowers (PL–68.5%) in population ISTb did not differ significantly from open pollinated flowers (C–53.2%), suggesting that this population is not pollen/pollinator limited. In addition, high gene flow was observed between individuals of adjacent sites of OSP (GL, CK) and ISTa (ISTb), the two largest and, according to our observations, densest populations of *M. tommasinii*. In contrast, the significantly smaller population PP, with individuals scattered over the site, is expected to suffer from pollen and/or pollinator limitation (e.g. [93]).

### Hybridization with a closely related taxon

*Moehringia tommasinii* may undergo enhanced selfing at sites where it closely co-occurs with the related *M. muscosa* to form a barrier to interspecific gene flow, as seen, for example, in *Caulophyllum* [94], *Mimulus* [95], *Clarkia* [96], and *Centaurium* [97–99]. Population genetic structuring, extensive gene flow and morphometric analyses revealed extensive hybridization followed by almost complete introgression at locality GL, where the taxonomic and genetic identity of *M. tommasinii* is nowadays severely questioned. Compared to other populations, overall low fruit set was obtained in the population GL regardless of pollination treatment, while spontaneous selfing (A_s_) did not produce fruit. The mating system of the population GL differed from that of the other populations by a significantly lower fruit set between treatments A_i_ (7.7%) and G (33.3%), while allogamous treatments produced a fruit set comparable to the fruit set of interspecific crosses between *M. tommasinii* and *M. muscosa* (30%).

The overall low fruit set, low seed germination, and low seedling survival in population GL suggest stronger postzygotic than prezygotic barriers in the studied system. The most effective prezygotic barrier is probably the ecological isolation of *M. tommasinii* from *M. muscosa*. Based on morphological evidence, spontaneous hybridization in nature had already been demonstrated between individuals of *M. muscosa* with tetramerous and *M. ciliata* with pentamerous flowers, resulting in *M. x hybrida* Kerner in Handel-Manzzetti, as well as between *M. muscosa* and *M. bavarica* (a species closely related to *M. tommasinii*) resulting in *M. x coronensis* Behrendsen, and have been reported several times in the overlapping range of the two taxa [58, 69, 100, 101]. A recent population genetic study of *Moehringia jankae* and *M. grisebachii* using ISSR markers has shown that in populations with similar conditions and geographic proximity, individuals of *M. grisebachii* show greater similarity in the genotype to co-occurring individuals of *M. jankae* than to allopatric conspecifics and that a plausible explanation is the possibility of hybridization between co-occurring populations of the two taxa [102]. Morphologically, the co-occurring individuals of *M. grisebachii* with hairy stems also have a less pronounced indumentum and are similar in appearance to individuals of *M. jankae*, which are glabrous (ibid.).

Hind [69] reported some teratological variation also in the flowers of *M. tommasinii* and the closely related *M. bavarica*, the latter collected from the type locality of the hybrid *M.* x *coronensis* (cliffs and walls of the monastery of Madonna della Corona, Italy) and showing a slightly diminutive flower form. On one hand, most flowers of *M. tommasinii* were tetramerous. No teratological aberrations were observed in outcrossing populations OSP and ISTb of *M. tommasinii*, while in populations VDC^m^ of *M. muscosa* aberration frequency was low. On the other hand, the highest variation in the number of stamens, and consequently the P/O ratio, was observed in population GL of *M. tommasinii*. These results are largely consistent with the observations of Rieseberg and Ellstrand [103], who found the highest proportion of “extreme” character values in hybrids.

Given the weak reproductive barriers between the species, these individuals may actually represent crosses and backcrosses between *M. muscosa* and *M. tommasinii* at various stages of introgression. The high plasticity of both reproductive and vegetative characters, coupled with hybridization and convergence of evolutionarily distinct lineages due to adaptations in morphology, anatomy, and physiology to specific environments, makes answering questions about the systematics of *Moehringia* particularly challenging [56, 58, 59, 69].

### Reproductive isolation and conservation issues

The results of population genetic structuring and gene flow were congruent. High and moderate gene flow between populations of *M. tommasinii* (GL) and *M. muscosa* (GL^m^ and VDC^m^) were reflected in low levels of genetic differentiation both within and between the species. In *M. tommasinii*, high gene flow was also observed between populations OSP–CK and ISTa–ISTb, which formed separate genetic clusters. On the other hand, population PP and the ISTa–ISTb population pair of *M. tommasinii* appeared to be the most reproductively isolated, forming distinct, genetically well-differentiated groups.

However, there was a discrepancy between the observed heterozygosity (*H*_*o*_) and allelic richness (*N*_*ar*_) values compared to the results obtained from the mating systems and morphometric analyses. Surprisingly, the population with the lowest observed heterozygosity was ISTb, which is one of the largest populations. Conversely, the population with the highest observed heterozygosity was GL, which is the smallest population. This incongruity suggests that additional factors may be at play influencing the genetic diversity and structure within these populations. Observed low to moderate levels of genetic variability were expected, as similar results can be found in studies of narrow-endemics (e.g. [104, 105]).

The number of detected private alleles was similar in most populations, with exception of the population PP and to a somewhat smaller extent, the population OSP. The substantially larger number of private alleles detected in these populations suggested their prolonged isolation and absence of inter-population gene flow, which resulted in the accumulation of population-specific genetic mutations.

In three populations of *M. tommasinii*, a slightly positive and significantly higher within-population fixation index (*F*_is_) was detected, suggesting pronounced breeding among closely related individuals, which is common in small populations. In addition, a significantly negative correlation between population sizes and their *F*_is_ value supported the expectation that smaller populations are more susceptible to increased levels of inbreeding than the larger ones [106]. However, it is important to highlight that the significant negative correlation between population size and *F*_is_ is primarily attributed to the ISTa population. In contrast, for the other populations, no definitive positive or negative correlation could be established between population size and *F*_is_. At this point, it should be noted that inbreeding here discussed represents the genetic status of the studied populations, while the inbreeding depression discussed as a part of the mating system analysis presents a population’s potential for a specific mating model. Depending on circumstances, this model can, but does not have to be expressed in a natural environment.

Knowing that *M. tommasinii* distribution range stretches for less than 30 km, it was interesting to see four well-defined genetic clusters across only six extant populations. Such a structuring likely emerged as a consequence of very limited current gene flow among most of the populations because of the strongly fragmented habitat this species inhabits. We see little consistency when comparing preferred mating systems of populations with their genetic clusters affiliations. For example: neighbouring populations CK and PP from close proximity (app. 2.6 km apart) belong to different genetic clusters but both prefer selfing over outcrossing. To that end, population PP is strongly genetically differentiated, show very limited gene exchange with neighbouring populations, and albeit being one of the smallest in number of individuals, it harbours the highest number of private alleles. There are two possible explanations for such an observation: (a) the preferred mating system is influenced by certain extrinsic factors, for example distinct pollinator assemblages, close proximity of *M. muscosa* and a shift in mating system from outcrossing to selfing, and (b) the system is regulated by the limited number of genetic elements that evolved independently from the rest of the genome and are under strong selection pressure. Unfortunately, our genetic analysis approach has low resolution, and we could not answer these questions.

Another important finding was the extensive hybridization between *M. tommasinii* and *M. muscosa* followed by introgression detected in population GL with further support in morphometrics. From the results obtained by Bayesian assignment test, it seems that the hybridization process has been active for a prolonged period, and probably extends for at least dozens of generations. This conclusion is based on the genetic composition of the studied individuals, because in all of them the genetic cluster of *M. muscosa* predominates over the cluster of OSP–CK, indicating that the individuals originate from a later backcross generation with *M. muscosa*. It also seemed that the hybrid offspring is fertile, and that there are no intrinsic reproduction barriers between these taxa. This is consistent with the results obtained from interspecific controlled cross-pollination and moderate gene flow between *M. muscosa* and *M. tommasinii*. Since it seems there are no effective interspecific reproduction barriers, a more abundant population assimilated the other one through the hybridization event.

Such a finding demonstrates the possible negative influence of spontaneous hybridization on narrowly distributed endangered taxa that can lead to extinction through assimilation [103, 107, 108]. Based on the clear evidence in the molecules and flower morphology, the population GL can hardly be attributed to *M. tommasinii* at present, but is almost completely assimilated by *M. muscosa*, leading to local extinction of *M. tommasinii*.

### Selfing syndrome

The occurrence of a selfing syndrome should allow maximizing the benefits of self-fertilization through reproductive assurance, transmission advantage, and gene purging while minimizing its negative consequences in the form of inbreeding depression and lower reproductive output [16]. This should require morphological, anatomical, and physiological components of the mating system to enhance autonomous selfing. Compared to allogamous treatments, rates of autonomous selfing in *M. tommasinii* were low in all populations. Smaller populations suffered from lower pollen production and seed weight. However, the increase of selfing in small populations appeared to be due to geitonogamy, which is traditionally considered an ineffective selfing mode because it requires pollen vectors and is thus subject to the same limitations as outcrossing. The functional limitations based on geitonogamous selfing, do not adequately support the hypothesis that lower pollen production and seed weight and pronounced selfing are the result of a selfing syndrome. The less conspicuous floral display in smaller populations with pronounced selfing of *M. tommasinii* is also not a consequence of the selfing syndrome, but rather due to fitness limitations. This suggests that *M. tommasinii* would not be functionally able to cope with increasing inbreeding with decreasing population size, as has been widely demonstrated for plants possessing cross-biased mating systems (e.g. [109, 110]).

## Conclusions

All studied populations of *Moehringia tommasinii* are self-compatible, with geitonogamous hand-pollination resulting in a relatively high fruit set. However, limited autonomous selfing suggests that assisted pollination is required for an effective pollination across the entire distribution range. Generally, population size proved to be the most important factor affecting the mating system in genetically structured populations of *M. tommasinii*. Less conspicuous floral display in smaller populations, coupled with lower pollen production and seed weight, are not due to a selfing syndrome but rather to a fitness limitation. Detected gene flow between *M. tommasinii* and the sympatric *M. muscosa* suggested weak reproductive barriers between the taxa. Hybridization may have significantly disrupted mating patterns of *M. tommasinii*, triggering floral polymorphism and causing local population extinction.

### Methods

#### Material sampling

In each population of *Moehringia tommasinii*, seeds and leaf tissue were carefully collected from 30 individuals to avoid damaging the plants. In total, 217 samples of leaf tissues were collected, 30 from each one of six *M. tommasinii* populations, and 16 and 21 from two selected *M. muscosa* populations, one being sympatric with *M. tommasinii*. Immediately after sampling, leaf tissue was stored in silica gel for rapid desiccation, while seeds were suitably dried and stored at − 20 ^0^ C in a refrigerator. In each population, we randomly selected a minimum of 16 to a maximum of 32 individuals and one bud and one freshly opened flower were collected from each individual and stored in a solution of 96% ethanol and glycerol (1:1) for later processing in the laboratory. Our preliminary observations indicated that variation in petal characteristics within the same plant was neglectable (unpublished data), thus, sampling a single flower per plant adequately represented the overall variability within and among populations. Flowers of *M. muscosa*, one in close sympatry with *M. tommasinii* (locality GL), were sampled from three populations and served as outgroups. The number of individuals per population was assessed by direct counting using binocular (Swarovski 10 × 25 CL pocket binocular) and a spotting scope (Kowa TSN-663 M).

### Seed germination and plant establishment

Seeds collected from randomly selected individuals (one seed per individual) in each population (30 seeds belonging to 30 individuals) were first weighted to the nearest 10 µg (Ohaus AP250D). To test the differences in seed weight among populations we used Kruskal–Wallis test for equal of medians, and Mann–Whitney pairwise comparisons (Bonferroni corrected p–values) as a post-hoc test, implemented in PAST [111]. We germinated seeds according to a pre-optimised protocol. Seeds were chemically pre-treated with gibberellic acid (GA_3_) and mechanically treated by removing the strophiole and scarifying the testa by making a small incision with a scalpel. Seeds were germinated on 1% agar at 20 °C with a photoperiod of 16 h of light and 8 h of darkness in growth chambers. Germination was checked every other day for approximately 2 months until germination reached a plateau. In late winter, the individual seedlings were transplanted into pots and raised in an open nursery at the Natural History Museum Rijeka to ensure equal growing conditions. The plants flowered profusely in spring of the same year. Fully grown plants, isolated from potential visitors/pollinators, were for most of the day sheltered from direct sun and rain.

### Flower life span and sexual functioning

Flower longevity was studied by marking 249 flowers on several individuals (one per individual) before opening. The flowers were then observed daily until senescence. We assessed pollen viability throughout the flower lifespan through diaminobenzidine reaction (DAB) [112], assuming that viable pollen, as opposed to dead and aborted pollen, stains dark reddish brown as a result of enzyme activity [113]. The efficacy of the stain was first tested on pollen killed by high temperature. At least 200 grains (and up to 400) per flower were counted when evaluating the percentage of stained pollen. Stigma receptivity throughout the life span of the flower was evaluated by counting pollen grains germinating on the stigma and the growth of pollen tubes along the style according to Vaughton and Ramsey [114]. Equal amounts of pollen from different individuals (3–5) were applied evenly on stigmatic lobes to avoid oversaturation. About 6 h later, the stigmas were collected, fixed in 70% ethanol, and mounted directly on the slide in a drop of 0.01% decolorized aniline blue, squashed and stained for 1 h. Samples were observed using an epifluorescence microscope (Nikon Eclipse 55i) with UV–1 A filter (360–370 nm). The number of pollen tubes penetrating the stigmatic papillae and style was recorded. A total of 108 and 141 flowers were collected from different individuals (one per individual) in all populations to evaluate pollen viability and stigma receptivity, respectively. For more details, see Fig. 2a. Since the datasets did not meet the assumptions on normality and homogeneity of variance, differences in pollen viability and stigma receptivity through a flower life-span was tested using the Kruskal–Wallis test for equality of medians and the Mann–Whitney pairwise comparison (Bonferroni-corrected p-values) post-hoc test implemented in R package ‘stats’ [115].

### Mating system and pollen limitation

The following treatments were applied to determine the mating system, effects of insect exclusion and pollen source on fruit production of *M. tommasinii*: (a) spontaneous autogamy (A_s_) – flowering plants were covered with a tulle to exclude insect interactions and left intact; (b) induced autogamy (A_i_) – flowering plants were covered with a tulle to exclude insect interactions and individual flowers were pollinated with their own pollen; (c) geitonogamy (G) – flowering plants were covered with a tulle and pollinated with pollen from flowers of the same plant; (d) xenogamy (Xe) – flowering plants were covered with a tulle and pollinated with a fresh pollen mixture collected from several individuals (3–5) from the same population; (e) between-population xenogamy (Xe_bp_) – flowering plants were covered with a tulle and pollinated with a fresh pollen mixture collected from several individuals (3–5) from different population; (f) supplementary pollination (PL) – flowers were pollinated in situ with a fresh pollen mixture collected from several individuals (3–5); (g) control (C) – flowers were labelled in situ and left intact. Treatments (a) – (e) were performed in the garden, as individuals from all but one population (ISTb) were inaccessible to manipulation in their natural sites in sufficient number. Treatments (f) and (g) were performed only in one population (ISTb) where a sufficient number of plants were accessible. Reciprocal crosses were performed among all populations, except for the population GL, where the number of crosses was generally lower due to the small number of individuals recruited from seeds and high seedling mortality. The sample size for all treatments is indicated in the Fig. 2b. Crosses with sympatric, closely related but ecologically divergent *Moehringia muscosa* were made on 18 flowers of *M. tommasinii* from GL, ISTa, ISTb, and CK on 4, 2, 11, and 1 flower, respectively, using *M. muscosa* as pollen donor. All pollination treatments involving controlled pollen transfer were performed *ex situ* with a stereomicroscope (Olympus SZX10) and in situ with a hand lens (40x) to ensure successful pollination. After 60 days, fruit and seed production (fully developed seeds with embryos) were recorded.

From the results of hand pollinations, we calculated two indices related to the mating system. To distinguish possible effects of dichogamy from genetic incompatibility, autogamous performance was determined from the results of geitonogamous pollen transfer. The degree of inbreeding depression (δ_i_ – inbreeding depression coefficient was determined by the ratio between fruit set and reproductive success of geitonogamously (*wG*) and xenogamously pollinated flowers (*wXe*), as suggested by Charlesworth and Charlesworth [67]: *δ*_*i*_ = 1 – (*wG* / *wXe*). On the other hand, outbreeding depression coefficient (*δ*_*o*_) indicates the degree of outbreeding depression as determined by the ratio of fruit set to reproductive success of xenogamously pollinated flowers between (wXe_bp_) and within populations (wXe): *δ*_*o*_ = 1 – (*wXe*_*bp*_ / *wXe*) [68]. The magnitude of inbreeding depression is negatively correlated with the population selfing preferences [116], where higher positive values of inbreeding and outbreeding coefficients indicate higher preferences to outcrossing and selfing, respectively, and vice versa. Reproductive success of each population after the different pollination treatments was calculated from fruit set, without considering herbivory rates, because plants were raised and manipulated in controlled environment protected from grazing.

Responses to each manipulation were determined using generalized linear models (GLM) with fruit set as the response variable and treatments and populations as predictors, considering all populations and each population separately. The response variable was fitted to a binomial distribution with a logit link function. Likelihood ratio test was performed to compare the full model with a restricted model and by calculating *p* values using the χ^2^ distribution. Differences between levels of each effect were analysed post hoc by multiple comparisons of means with Tukey contrasts, adjusting data for normality and testing for homogeneity of variance. Statistical analyses were performed using the R packages ‘stats’, ‘car’, and ‘multcomp’ [115].

### Flower morphology and pollen-to-ovule ratio

To test for a possible selfing syndrome, beside seed weight (see above), flower size and the number of pollen grains and ovules per flower were determined. Petal width and length of 16–32 flowers per population of *M. tommasinii* and *M. muscosa* were measured using ImageJ software [117] and a simple petal index (petal width/length) was calculated. The number of flowers measured per population is indicated in the Fig. 6a. Since the dataset did not meet the assumptions on normality and homogeneity of variance, statistical significance of differences among samples was tested using the Kruskal–Wallis test for equal medians and the Mann–Whitney test for pairwise comparisons as a post hoc test with PAST. Results were considered significant if the probability of the null hypothesis was less than 0.05 (Bonferroni corrected *p*–values). The ovules per flower were counted under a dissecting microscope. To estimate the number of pollen grains per flower, all anthers of flower buds were cut and placed in a 2 mL microcentrifuge tube containing 1 ml acetic acid and stored at 5 °C for one week. To prepare the pollen for counting, the tubes containing the partially dissolved anther tissue were vortexed and put in a microcentrifuge at 10,000 rpm for 3 min. After decanting the supernatant, the pollen samples were repeatedly washed with 1.5 mL of ethyl alcohol. At the end, the ethyl alcohol in the tubes was left to evaporate, and the tubes were refilled with 50 µL of distilled water. To ensure that the pollen remained homogeneously suspended in the solution, the tube was thoroughly vortexed before the sample was pipetted onto a haemocytometer (Improved Neubauer), where individual pollen grains were counted in a chamber of known volume. The total number of pollen grains per flower was then adequately calculated. Pollen grains and ovules were counted for each population of *M. tommasinii* and one population of *M. muscosa* (population VDC^m^). In addition, the pollen-to-ovule ratio (P/O ratio: the total number of pollen grains produced per flower divided by the number of ovules) was calculated as a proxy for the mating system [118]. Petal polymorphism and variation in P/O ratio within and among populations of *M. tommasinii* and *M. muscosa* were assessed using the Kruskal–Wallis test for equality of medians and the Mann–Whitney pairwise comparison (Bonferroni-corrected *p*–values) post-hoc test since the data did not meet the assumptions on normality and homogeneity of variance.

#### DNA extraction, development of molecular markers and population genetics

DNA from leaf tissues was extracted using a GenElute plant genomic DNA miniprep kit following the given instructions (Sigma-Aldrich, St. Louis, MO, USA).

Since no microsatellite molecular markers were available for *Moehringia tommasinii* or any closely related species, a set of microsatellite markers was developed so that reliable assessment of the species’ population-genetic structure could be performed. After the sampling, the leaf tissue of collected individuals was immediately stored in silica gel for rapid desiccation. Genomic DNA was extracted using GenElute™ Plant Genomic DNA Miniprep Kit (Sigma-Aldrich®). DNA isolates were submitted to the AllGenetics & Biology SL (A Coruña, Spain) for the library development, sequencing, and loci characterization. For the development of the microsatellite library, an individual from the population CK was sequenced, while additional 48 samples from different populations of both studied species were used for loci characterization. Voucher specimen is deposited at the herbarium of the Natural History Museum Rijeka (NHMR 3164). A library was prepared using the Nextera XT DNA kit (Illumina), following the manufacturer’s instructions. The library was enriched by hybridization with the AC, AG, ACG, and ATCT microsatellite motifs. The sequencing was performed using a 2 × 150 paired-end protocol on an Illumina MiSeq (Illumina, San Diego, California, USA). CLC Genomics Server software (ver. 10.0.1) was used for removal of the adapter sequences and trimming, followed by the de novo assembly. QDD 3.1 software was employed for the identification of microsatellite regions within the assembled contigs. Primer pairs were developed with the Primer3 program [119] as implemented in QDD 3.1 software [120]. Default settings were used, with product size range 100–300 bp, GC content between 30–70%, and melting temperature (Tm) between 57–62 °C. Only loci with pure microsatellites were considered for primer development. For initial loci characterization, PCR testing of 48 selected primer pairs was performed on five randomly selected *M. tommasinii* and *M. muscosa* individuals. Before the amplification of the entire sample set of 216 studied individuals, ten loci characterized by optimal amplification patterns and satisfying polymorphism levels were selected and additionally tested on 24 individuals from both studied species. All PCR reactions were carried out following Schuelke [121], which implies the usage of a fluorescently-labelled oligonucleotide identical to the 5’ tail of the reverse primer. The oligonucleotide tails used were the universal sequences M13 (GGA AAC AGC TAT GAC CAT), CAG (CAG TCG GGC GTC ATC), and T3 (AAT TAA CCC TCA CTA AAG GG). The three oligonucleotides were labelled with the HEX dye, the FAM dye, and the TAMRA dye, respectively. Loci amplifications were carried out on the GenAmp® PCR System 9700 (Applied Biosystems, Foster City, CA, USA) using a two-step protocol with an initial touchdown cycle with following cycling conditions: 94 °C for 5 min; five cycles of 45 s at 94 °C, 30 s at 60 °C for the first cycle and 1 °C less in each subsequent cycle, and 90 s at 72 °C; 25 cycles of 45 s at 94 °C, 30 s at 55 °C, and 90 s at 72 °C; and an 8 min extension step at 72 °C. Finally, obtained PCR products were run on an ABI 3730XL (Applied Biosystems, Foster City, CA, USA) and the results were analysed and scored using GeneMapper 4.0 software (Applied Biosystems, Foster City, CA, USA).

For each microsatellite locus, basic population-genetic parameters (number of alleles per locus, the observed heterozygosity, the expected heterozygosity, and the polymorphic information content) were calculated using Cervus 3.0.7 software [122]. The obtained summary statistics of loci selected for population-genetic analysis is given in Additional file 8: Table S8. To estimate basic population-genetic parameters (observed and expected heterozygosity, and within-population fixation index), we used GENEPOP software [123]. We used the R package “PopGenReport” [124] to calculate allelic richness (*N*_ar_) and to construct the pairwise *F*_st_ distance matrix, while the R package “poppr” [125] was used for the estimation of the private alleles number. Gene flow between populations (*N*_m_) was calculated from the pairwise *F*_st_ values using the formula: *N*_m_ = (1 − *F*_st_) / 4 *F*_st_ [126]. To assess the distribution of the individuals from different populations, we performed the allelic frequencies-based principal component analysis (PCA) as implemented in the R package “adegenet” [127]. The same software was used for the construction of the Nei’s [128, 129] genetic distance matrix which was then used for the reconstruction of the neighbor-joining network using SplitsTree4 software [130]. Bootstrap support was obtained using 1,000 replicates generated by R package “poppr”. To assess the genetic structure of the populations studied, the Bayesian assignment test was performed using the software STRUCTURE ver. 2.3.3 [131]. Fifteen runs per cluster (K) were performed, with K ranging from 1 to 9. A mixture model and correlated allele frequencies were assumed, with no prior information on the origin of the population. Each run consisted of a burn-in period of 200,000 steps followed by 1.000,000 MCMC replicates. Fifteen runs per cluster (*K*), with *K* ranging from 1 to 9, were carried out, assuming an admixture model and correlated allele frequencies, with no prior information on population origin. Each run consisted of a burn-in period of 200 000 steps followed by 1,000,000 MCMC replicates. STRUCTURE HARVESTER v0.6.92 [132]. was used to process the results of Bayesian assignment test. By comparing the average estimates of the likelihood of the data, *ln[Pr(X*|*K)]*, for each value of *K*, as well as by calculating an ad hoc statistic Δ*K* based on the rate of change in the log probability of data between successive *K* values [133], the choice of the most likely number of clusters (*K*) was made. Runs were clustered and averaged using CLUMPAK [134]. The correlation between the geographic distance matrix and the genetic similarity matrix was measured using Mantel tests in the R package [135]; a *p* value was obtained by 9,999 randomizations, with the null hypothesis of no relationship between the two matrices being true.

The relationships between the within-population fixation index (*F*_is_), floral display size (petal width/petal length), seed weight, fruit production by outcrossing (xenogamy), and population size on the one hand, and the within-population fixation index, outcrossing, and population size as factors on the other, were analysed separately for all populations using the same procedure. Response variables (floral display, pollen-to-ovule ratio, seed weight) were adjusted to a normal distribution with identity link function using algorithms implemented in R package ‘stat’ to model responses. The significance of slope was estimated using the G–statistics, an equivalent to χ^2^, with one degree of freedom.

### Supplementary Information

**Additional file 1.**

**Additional file 2.**

**Additional file 3.**

**Additional file 4.**

**Additional file 5.**

**Additional file 6.**

**Additional file 7.**

**Additional file 8.**

**Additional file 9.**

## Data Availability

The datasets supporting the conclusions of this article are available from the corresponding author on reasonable request.
