# Supplementary material for: Population size as a major determinant of mating system and population genetic differentiation in a narrow endemic chasmophyte
Source: BMC Plant Biol. 2023 Aug 9;23:383. doi: 10.1186/s12870-023-04384-8 (PMC10411015; doi:10.1186/s12870-023-04384-8)
Supplement: Supplementary file 1 — Additional file 1. [file 12870_2023_4384_MOESM1_ESM.docx]

**Additional file 1**

**Table S1** Generalized linear modelling of controlled hand pollination treatments with *Moehringia tommasinii* drawn across all six populations – multiple comparisons of means (Tukey Contrasts).

|  | A_s_ | A_i_ | G | Xe | Xe_bp_ |
| --- | --- | --- | --- | --- | --- |
| A_s_ |  | <0.001 | <0.001 | <0.001 | <0.001 |
| A_i_ | 5.659 |  | 0.0587 | <0.001 | <0.001 |
| G | 6.852 | 2.622 |  | 0.1713 | 0.6369 |
| Xe | 7.722 | 4.650 | 2.175 |  | 0.7710 |
| Xe_bp_ | 7.445 | 4.136 | 1.350 | 0.7710 |  |

Lower left handed corner – z values, upper right handed corner – p-values. A_s_ – spontaneous selfing, A_i_ – induced selfing, G – geitonogamy, Xe – xenogamy, Xe_bp_ – between population crosses.
