# Supplementary material for: Population size as a major determinant of mating system and population genetic differentiation in a narrow endemic chasmophyte
Source: BMC Plant Biol. 2023 Aug 9;23:383. doi: 10.1186/s12870-023-04384-8 (PMC10411015; doi:10.1186/s12870-023-04384-8)
Supplement: Supplementary file 2 — Additional file 2. [file 12870_2023_4384_MOESM2_ESM.docx]

**Additional file 2**

**Table S2** Generalized linear modelling of controlled hand pollination treatments with *Moehringia tommasinii* for populations GL, OSP, CK, PP, ISTa and ISTb – multiple comparisons of means (Tukey Contrasts).

|  | **GL** (χ^2^ = 20.25, df .= 4, p < 0.0001) | | | | |  |  | **OSP** (χ^2^ = 61.76, df. = 4, p < 0.0001) | | | | |  |  | **CK** (χ^2^ = 24.44, df. = 4, p < 0.0001) | | | | |  |  |
| --- | --- | --- | --- | --- | --- | --- | --- | --- | --- | --- | --- | --- | --- | --- | --- | --- | --- | --- | --- | --- | --- |
|  | **A_s_** | **A_i_** | **G** | **Xe** | **Xe_bp_** | **PL** | **C** | **A_s_** | **A_i_** | **G** | **Xe** | **Xe_bp_** | **PL** | **C** | **A_s_** | **A_i_** | **G** | **Xe** | **Xe_bp_** | **PL** | **C** |
| **PLA_s_** |  | 1 | 1 | 1 | 1 | . |  |  | 1 | 1 | 1 | 1 | . | . |  | **0.0394** | **0.001** | **0.0323** | 0.1471 | . | . |
| **A_i_** | 0.012 |  | 0.619 | 0.319 | 0.418 | . | . | 0.020 |  | 0.991 | **0.055** | **p<0.001** | . | . | 2.778 |  | 0.9157 | 1 | 0.8088 | . | . |
| **G** | 0.014 | 1.323 |  | 1 | 1 | . | . | 0.020 | 0.423 |  | 0.092 | **p<0.001** | . | . | 3.222 | 0.831 |  | 0.936 | 0.2534 | . | . |
| **Xe** | 0.014 | 1.790 | 0.194 |  | 1 | . | . | 0.022 | 2.580 | 2.383 |  | 0.696 | . | . | 2.849 | 0.092 | -0.766 |  | 0.7317 | . | . |
| **Xebp** | 0.014 | 1.622 | 0.087 | -0.152 |  | . | . | 0.023 | 3.942 | 4.002 | 1.212 |  | . | . | 2.595 | -1.075 | -1.994 | -1.211 |  | . | . |
| **PL** | . | . | . | . | . |  | . | . | . | . | . | . |  | . | . | . | . | . | . |  | . |
| **C** | . | . | . | . | . | . |  | . | . | . | . | . | . |  | . | . | . | . | . | . |  |
|  | **PP** (χ^2^ = 49.08, df. = 4, p < 0.0001) | | | | |  |  | **ISTa** (χ^2^ = 12.297, df. = 2, p = 0.0021) | | | | |  |  | **ISTb** (χ^2^ = 87.229, df. = 6, p < 0.0001) | | | | |  |  |
| **A_s_** |  | **p<0.01** | **p<0.005** | **0.0156** | 0.1868 | . | . |  | 1 | 1 | 1 | 1 | . | . |  | **0.0356** | **p<0.01** | **p<0.001** | **p<0.001** | **p<0.001** | **p<0.01** |
| **A_i_** | 3.388 |  | 0.9840 | 0.9856 | **0.0692** | . | . | 0.014 |  | 0.2582 | **p<0.005** | **p<0.001** | . | . | 3.018 |  | 0.9113 | **p<0.05** | **p<0.05** | 0.2609 | 0.9813 |
| **G** | 3.797 | 0.523 |  | 0.8076 | **p<0.005** | . | . | 0.015 | 1.922 |  | 0.46203 | 0.31804 | . | . | 3.568 | 1.125 |  | **p<0.05** | 0.2609 | 0.9405 | 0.9989 |
| **Xe** | 3.092 | -0.508 | -1.079 |  | 0.23964 | . | . | 0.015 | 3.266 | 1.570 |  | 0.99994 | . | . | 4.992 | 3.726 | 2.949 |  | 0.8666 | 0.1931 | **p<0.005** |
| **Xebp** | 2.151 | -2.576 | -3.498 | -2.026 |  | . | . | 0.015 | 3.527 | 1.807 | 0.120 |  | . | . | 4.599 | 3.243 | 2.221 | -1.238 |  | 0.7685 | **p<0.05** |
| **PL** | . | . | . | . | . |  | . | . | . | . | . | . |  | . | 4.076 | 2.222 | 1.031 | 2.368 | 1.427 |  | 0.5451 |
| **C** | . | . | . | . | . | . |  | . | . | . | . | . | . |  | 3.464 | 0.814 | 0.485 | 3.472 | 2.962 | 1.765 |  |

Lower left handed corner – z values, upper right handed corner – p-values. A_s_ – spontaneous selfing, A_i_ – induced selfing, G – geitonogamy, Xe – xenogamy, Xe_bp_ – between population crosses, PL – pollen limitation, C – control. Red color indicates near statistical significance.
