# Supplementary material for: Population size as a major determinant of mating system and population genetic differentiation in a narrow endemic chasmophyte
Source: BMC Plant Biol. 2023 Aug 9;23:383. doi: 10.1186/s12870-023-04384-8 (PMC10411015; doi:10.1186/s12870-023-04384-8)
Supplement: Supplementary file 3 — Additional file 3. [file 12870_2023_4384_MOESM3_ESM.docx]

**Additional file 3**

**Table S3** Generalized linear modelling of controlled hand pollination treatments with *Moehringia tommasinii* for populations GL, OSP, CK, PP. ISTa and ISTb – multiple comparisons of means (Tukey Contrasts).

|  |  |  |  | **A_s_** |  |  |  |  |  | **A_i_** |  |  |  |  |  | **G** |  |  |  |  |  | **Xe** |  |  |  |  |  | **Xe_bp_** |  |  |  |  |  | **PL** | **C** | **MtxMm** |
| --- | --- | --- | --- | --- | --- | --- | --- | --- | --- | --- | --- | --- | --- | --- | --- | --- | --- | --- | --- | --- | --- | --- | --- | --- | --- | --- | --- | --- | --- | --- | --- | --- | --- | --- | --- | --- |
| **Manipulation** | **Populations** | **% fruit set** | **no. manipulations** | **GL** | **OSP** | **CK** | **PP** | **ISTa** | **ISTb** | **GL** | **OSP** | **CK** | **PP** | **ISTa** | **ISTb** | **GL** | **OSP** | **CK** | **PP** | **ISTa** | **ISTb** | **GL** | **OSP** | **CK** | **PP** | **ISTa** | **ISTb** | **GL** | **OSP** | **CK** | **PP** | **ISTa** | **ISTb** | **ISTb** | **ISTb** |  |
| **A_s_** | GL | 0 | 25 |  | / | 0,3711 | 0,1914 | / | 0,3944 | 0,1653 | 0,0606 | 0,0004 | <0,0001 | 0,0255 | 0,0002 | 0,0015 | 0,0307 | <0,0001 | <0,0001 | 0,0004 | <0,0001 | 0,0006 | 0,0001 | 0,0003 | 0,0001 | <0,0001 | <0,0001 | 0,0015 | <0,0001 | 0,0042 | 0,0034 | <0,0001 | <0,0001 | <0,0001 | <0,0001 | 0,0038 |
|  | OSP | 0 | 25 | / |  | 0,3711 | 0,1914 | / | 0,3944 | 0,1653 | 0,0606 | 0,0004 | <0,0001 | 0,0255 | 0,0002 | 0,0015 | 0,0307 | <0,0001 | <0,0001 | 0,0004 | <0,0001 | 0,0006 | 0,0001 | 0,0003 | 0,0001 | <0,0001 | <0,0001 | 0,0015 | <0,0001 | 0,0042 | 0,0034 | <0,0001 | <0,0001 | <0,0001 | <0,0001 | 0,0038 |
|  | CK | 3,2 | 31 | 0,8 | 0,8 |  | 0,5309 | 0,4015 | 0,944 | 0,5174 | 0,1532 | 0,0005 | <0,0001 | 0,0566 | 0,0002 | 0,0023 | 0,0689 | <0,0001 | <0,0001 | 0,0004 | <0,0001 | 0,0008 | 0,0001 | 0,0003 | 0,0001 | <0,0001 | <0,0001 | 0,0029 | <0,0001 | 0,005 | 0,0071 | <0,0001 | <0,0001 | <0,0001 | <0,0001 | 0,0079 |
|  | PP | 6,7 | 30 | 1,707 | 1,707 | 0,39 |  | 0,2201 | 0,4719 | 0,9071 | 0,3982 | 0,0025 | 0,0001 | 0,1747 | 0,0012 | 0,01 | 0,2066 | 0,0001 | <0,0001 | 0,0021 | <0,0001 | 0,004 | 0,0007 | 0,0015 | 0,0004 | <0,0001 | <0,0001 | 0,0132 | <0,0001 | 0,0282 | 0,0194 | <0,0001 | <0,0001 | <0,0001 | <0,0001 | 0,0323 |
|  | ISTa | 0 | 22 | / | / | 0,7 | 1,504 |  | 0,4244 | 0,1931 | 0,0779 | 0,0008 | <0,0001 | 0.0357 | 0,0004 | 0,0028 | 0,0421 | 0,0001 | <0,0001 | 0.0007 | <0,0001 | 0,0013 | 0,0003 | 0,0005 | 0,0002 | <0,0001 | <0,0001 | 0,0028 | <0,0001 | 0,007 | 0,0058 | <0,0001 | <0,0001 | <0,0001 | <0,0001 | 0,0064 |
|  | ISTb | 2,9 | 35 | 0,725 | 0,725 | 0,005 | 0,518 | 0,638 |  | 0,466 | 0,1198 | 0,0002 | <0,0001 | 0,0397 | 0,0001 | 0,0011 | 0,0493 | <0,0001 | <0,0001 | 0,0002 | <0,0001 | 0,0004 | 0,0001 | 0,0001 | <0,0001 | <0,0001 | <0,0001 | 0,0015 | <0,0001 | 0,004 | 0,0025 | <0,0001 | <0,0001 | <0,0001 | <0,0001 | 0,0045 |
| **A_i_** | GL | 7,7 | 13 | 1,925 | 1,925 | 0,419 | 0,014 | 1,694 | 0,531 |  | 0,6028 | 0,0351 | 0,0055 | 0,3775 | 0,0237 | 0,079 | 0,4167 | 0,0067 | 0,0012 | 0,0347 | 0,0024 | 0,0481 | 0,0177 | 0,028 | 0,0138 | 0,002 | <0,0001 | 0,0816 | 0,0004 | 0,1407 | 0,1293 | 0,0009 | <0,0001 | 0,0001 | 0,0029 | 0,1365 |
|  | OSP | 13,3 | 30 | 3,52 | 3,52 | 2,04 | 0,714 | 3,108 | 2,421 | 0,271 |  | 0,02 | 0,0011 | 0,5979 | 0,0105 | 0,0661 | 0,6741 | 0,0014 | 0,0001 | 0,0176 | 0,0003 | 0,0308 | 0,0071 | 0,0135 | 0,0044 | 0,0002 | <0,0001 | 0,0797 | <0,0001 | 0,1545 | 0,122 | <0,0001 | <0,0001 | <0,0001 | 0,0003 | 0,1623 |
|  | CK | 40,7 | 27 | 12,653 | 12,653 | 12,168 | 9,168 | 11,307 | 13,724 | 4,439 | 5,41 |  | 0,3457 | 0,0564 | 0,844 | 0,5602 | 0,0404 | 0,4081 | 0,1299 | 0,9231 | 0,1965 | 0,8034 | 0,6891 | 0,9269 | 0,6356 | 0,1868 | <0,0001 | 0,7232 | 0,0815 | 0,2863 | 0,1812 | 0,1246 | 0,0005 | 0,0171 | 0,2809 | 0,4699 |
|  | PP | 53,3 | 30 | 18,445 | 18,445 | 18,737 | 15,253 | 16,607 | 20,915 | 7,707 | 10,625 | 0,889 |  | 0,0038 | 0,4422 | 0,1148 | 0,0023 | 0,8873 | 0,603 | 0,2601 | 0,7336 | 0,2153 | 0,5994 | 0,3767 | 0,6174 | 0,7507 | 0,0005 | 0,2397 | 0,5553 | 0,0362 | 0,0089 | 0,6484 | 0,0106 | 0,1692 | 0,9928 | 0,1197 |
|  | ISTa | 18,2 | 33 | 4,988 | 4,988 | 3,635 | 1,842 | 4,413 | 4,231 | 0,779 | 0,278 | 3,641 | 8,384 |  | 0,0315 | 0,1666 | 0,906 | 0,0048 | 0,0003 | 0,0515 | 0,0011 | 0,0846 | 0,0215 | 0,0396 | 0,0141 | 0,0007 | <0,0001 | 0,1844 | <0,0001 | 0,3489 | 0,3065 | 0,0002 | <0,001 | <0,0001 | 0,001 | 0,3386 |
|  | ISTb | 43,3 | 30 | 13,915 | 13,915 | 13,649 | 10,538 | 12,454 | 15,358 | 5,115 | 6,542 | 0,039 | 0,591 | 4,626 |  | 0,4217 | 0,0215 | 0,5185 | 0,1777 | 0,7537 | 0,2607 | 0,6444 | 0,8292 | 0,9127 | 0,7777 | 0,2514 | <0,0001 | 0,5952 | 0,1179 | 0,1916 | 0,0996 | 0,1742 | 0,0008 | 0,025 | 0,3759 | 0,3639 |
| **G** | GL | 33,3 | 32 | 10,06 | 10,06 | 9,316 | 6,631 | 8,958 | 10,579 | 3,086 | 3,377 | 0,339 | 2,487 | 1,913 | 0,646 |  | 0,1281 | 0,1409 | 0,0265 | 0,5945 | 0,051 | 0,7274 | 0,3209 | 0,4844 | 0,2708 | 0,0435 | <0,0001 | 0,8893 | 0,0095 | 0,6251 | 0,5132 | 0,0209 | <0,0001 | 0,0016 | 0,0682 | 0,8123 |
|  | OSP | 17,1 | 35 | 4,67 | 4,67 | 3,309 | 1,595 | 4,13 | 3,865 | 0,659 | 0,177 | 4,201 | 9,314 | 0,014 | 5,282 | 2,315 |  | 0,0029 | 0,0002 | 0,0359 | 0,0006 | 0,0618 | 0,0145 | 0,0275 | 0,0091 | 0,0004 | <0,0001 | 0,1477 | <0,0001 | 0,284 | 0,2383 | 0,0001 | <0,0001 | <0,0001 | 0,0005 | 0,2834 |
|  | CK | 51,5 | 33 | 17,897 | 17,897 | 18,166 | 14,734 | 16,098 | 20,302 | 7,349 | 10,159 | 0,684 | 0,02 | 7,936 | 0,417 | 2,168 | 8,852 |  | 0,4914 | 0,3142 | 0,6214 | 0,26 | 0,6885 | 0,4454 | 0,7129 | 0,631 | 0,0003 | 0,2811 | 0,4267 | 0,0455 | 0,0116 | 0,5226 | 0,0058 | 0,1149 | 0,8751 | 0,1432 |
|  | PP | 59,5 | 42 | 23,369 | 23,369 | 24,323 | 17,318 | 21,374 | 26,989 | 10,493 | 15,316 | 2,294 | 0,271 | 13,055 | 1,817 | 4,926 | 14,088 | 0,474 |  | 0,0758 | 0,8691 | 0,0625 | 0,2881 | 0,1395 | 0,2845 | 0,8245 | 0,0016 | 0,0946 | 0,9821 | 0,0053 | 0,0004 | 0,9022 | 0,0277 | 0,3629 | 0,5276 | 0,0378 |
|  | ISTa | 39,5 | 38 | 12,758 | 12,758 | 12,446 | 9,47 | 11,396 | 14,05 | 4,459 | 5,632 | 0,009 | 1,268 | 3,791 | 0,098 | 0,283 | 4,401 | 1,013 | 3,152 |  | 0,1306 | 0,865 | 0,5969 | 0,8411 | 0,5361 | 0,1181 | <0,0001 | 0,7691 | 0,0346 | 0,2909 | 0,1687 | 0,066 | 0,0001 | 0,006 | 0,1854 | 0,4942 |
|  | ISTb | 57,6 | 33 | 21,05 | 21,05 | 21,674 | 18,022 | 19,012 | 24,103 | 9,243 | 13,094 | 1,668 | 0,116 | 10,718 | 1,265 | 3,807 | 11,809 | 0,244 | 0,027 | 2,286 |  | 0,1075 | 0,388 | 0,2129 | 0,3915 | 0,9656 | 0,0014 | 0,1392 | 0,8618 | 0,0129 | 0,0018 | 0,9472 | 0,0243 | 0,3058 | 0,6832 | 0,0619 |
| **Xe** | GL | 37,5 | 32 | 11,667 | 11,667 | 11,137 | 8,263 | 10,411 | 12,595 | 3,907 | 4,662 | 0,062 | 1,536 | 2,975 | 0,213 | 0,121 | 3,49 | 1,269 | 3,469 | 0,029 | 2,591 |  | 0,4973 | 0,7111 | 0,4403 | 0,0936 | <0,0001 | 0,8911 | 0,0279 | 0,4064 | 0,2839 | 0,0523 | 0,0001 | 0,005 | 0,1458 | 0,6063 |
|  | OSP | 46,2 | 26 | 14,812 | 14,812 | 14,587 | 11,372 | 13,274 | 16,38 | 5,63 | 7,255 | 0,16 | 0,276 | 5,284 | 0,047 | 0,985 | 5,974 | 0,161 | 1,128 | 0,28 | 0,745 | 0,461 |  | 0,7467 | 0,958 | 0,38 | 0,0001 | 0,4839 | 0,2257 | 0,1384 | 0,0664 | 0,2963 | 0,0025 | 0,0567 | 0,5512 | 0,2855 |
|  | CK | 41,9 | 31 | 13,395 | 13,395 | 13,077 | 10,021 | 11,98 | 14,732 | 4,828 | 6,107 | 0,008 | 0,781 | 4,235 | 0,012 | 0,489 | 4,861 | 0,582 | 2,183 | 0,04 | 1,551 | 0,137 | 0,104 |  | 0,692 | 0,2023 | <0,0001 | 0,6581 | 0,0852 | 0,229 | 0,1215 | 0,1332 | 0,0005 | 0,0171 | 0,3068 | 0,4117 |
|  | PP | 46,9 | 32 | 15,554 | 15,554 | 15,619 | 12,362 | 14,025 | 17,521 | 6,06 | 8,093 | 0,225 | 0,25 | 6,023 | 0,08 | 1,213 | 6,797 | 0,135 | 1,145 | 0,383 | 0,734 | 0,595 | 0,003 | 0,157 |  | 0,3872 | 0,0001 | 0,4395 | 0,2131 | 0,1056 | 0,0409 | 0,2903 | 0,0018 | 0,049 | 0,5647 | 0,248 |
|  | ISTa | 57,1 | 42 | 21,901 | 21,901 | 22,707 | 19,003 | 17,503 | 25,247 | 9,595 | 13,936 | 1,743 | 0,101 | 11,498 | 1,316 | 4,076 | 12,683 | 0,231 | 0,049 | 2,443 | 0,002 | 2,812 | 0,755 | 1,626 | 0,748 |  | 0,0008 | 0,1326 | 0,8053 | 0,0097 | 0,0009 | 0,9025 | 0,0157 | 0,2522 | 0,6964 | 0,0564 |
|  | ISTb | 91,4 | 35 | 48,131 | 48,131 | 50,379 | 45,74 | 45,042 | 54,219 | 30,256 | 39,268 | 18,125 | 11,945 | 36,372 | 17,265 | 24,025 | 38,369 | 13,226 | 9,966 | 21,108 | 10,196 | 21,346 | 14,914 | 18,276 | 15,571 | 11,167 |  | <0,0001 | 0,0004 | <0,0001 | <0,0001 | 0,0006 | 0,21 | 0,012 | 0,0001 | <0,0001 |
| **Xe_bp_** | GL | 35,3 | 17 | 10,051 | 10,051 | 8,9 | 6,144 | 8,943 | 10,093 | 3,032 | 3,071 | 0,125 | 1,382 | 1,762 | 0,282 | 0,019 | 2,096 | 1,162 | 2,794 | 0,086 | 2,187 | 0,019 | 0,49 | 0,196 | 0,597 | 2,262 | 17,944 |  | 0,064 | 0,5825 | 0,4985 | 0,0919 | 0,0006 | 0,0153 | 0,1938 | 0,7417 |
|  | OSP | 59,3 | 113 | 28,61 | 28,61 | 30,506 | 26,084 | 25,712 | 33,994 | 12,397 | 19,926 | 3,034 | 0,348 | 17,14 | 2,445 | 6,731 | 18,911 | 0,632 | 0,001 | 4,463 | 0,03 | 4,833 | 1,468 | 2,962 | 1,55 | 0,061 | 12,394 | 3,431 |  | 0,001 | <0,0001 | 0,8969 | 0,0098 | 0,2527 | 0,4368 | 0,0207 |
|  | CK | 27,8 | 36 | 8,178 | 8,178 | 7,236 | 4,817 | 7,264 | 8271 | 2,17 | 2,028 | 1,137 | 4,389 | 0,877 | 1,705 | 0,239 | 1,148 | 4,001 | 7,774 | 1,116 | 6,183 | 0,689 | 2,196 | 1,147 | 2,619 | 6,682 | 29,29 | 0,302 | 10,776 |  | 0,9456 | 0,0034 | <0,0001 | 0,0002 | 0,0151 | 0,8672 |
|  | PP | 27,2 | 92 | 8,576 | 8,576 | 7,943 | 5,467 | 7,6 | 9,113 | 2,301 | 2,392 | 1,787 | 6,849 | 1,046 | 2,711 | 0,428 | 1,39 | 6,375 | 12.764 | 1,894 | 9,759 | 1,148 | 3,369 | 2,398 | 4,181 | 11,03 | 41,909 | 0,458 | 21,019 | 0,005 |  | 0,0001 | <0,0001 | <0,0001 | 0,0011 | 0,809 |
|  | ISTa | 58,3 | 66 | 24,975 | 24,975 | 26,261 | 22,283 | 22,533 | 29,187 | 10,982 | 16,755 | 2,358 | 0,208 | 14,153 | 1,847 | 5,333 | 15,598 | 0,409 | 0,015 | 3,379 | 0,004 | 3,766 | 1,091 | 2,254 | 1,118 | 0,015 | 11,763 | 2,841 | 0,017 | 8,604 | 15,365 |  | 0,0123 | 0,2519 | 0,563 | 0,0342 |
|  | ISTb | 81,4 | 43 | 41,319 | 41,319 | 43,499 | 38,91 | 38,206 | 47,226 | 23,196 | 32,483 | 12,018 | 6,535 | 29,624 | 11,234 | 17,585 | 31,583 | 7,623 | 4,85 | 14,808 | 5,076 | 15,034 | 9,113 | 12,164 | 9,698 | 5,838 | 1,571 | 11,767 | 6,679 | 22,674 | 34,603 | 6,266 |  | 0,1512 | 0,0031 | 0,0001 |
| **PL** | ISTb | 68,5 | 54 | 31,796 | 31,796 | 33,575 | 29,261 | 28,973 | 36,93 | 15,541 | 23,24 | 5,69 | 1,89 | 20,494 | 5,022 | 9,959 | 22,216 | 2,485 | 0,828 | 7,55 | 1,049 | 7,882 | 3,631 | 5,684 | 3,876 | 1,311 | 6,316 | 5,882 | 1,309 | 14,181 | 32,591 | 1,313 | 2,06 |  | 0,0944 | 0,0043 |
| **C** | ISTb | 53,2 | 62 | 21,175 | 21,175 | 22,043 | 18,346 | 19,042 | 24,607 | 8,858 | 13,245 | 1,163 | 0 | 10,788 | 0,784 | 3,325 | 12,005 | 0,025 | 0,399 | 1,754 | 0,167 | 2,116 | 0,355 | 1,044 | 0,332 | 0,152 | 14,609 | 1,689 | 0,605 | 5,906 | 10,595 | 0,335 | 8,762 | 2,799 |  | 0,0848 |
| **MtxMm** |  | 30 | 18 | 8,378 | 8,378 | 7,063 | 4,581 | 7,439 | 8,051 | 2,217 | 1,952 | 0,522 | 2,421 | 0,916 | 0,824 | 0,056 | 1,151 | 2,143 | 4,314 | 0,467 | 3,486 | 0,266 | 1,141 | 0,674 | 1,335 | 3,641 | 21,16 | 0,107 | 5,351 | 0,028 | 0,058 | 4,486 | 14,743 | 8,15 | 2,971 |  |

Lower left handed corner – χ^2^ values, upper right handed corner – p-values. A_s_ – spontaneous selfing, A_i_ – induced selfing, G – geitonogamy, Xe – xenogamy, Xe_bp_ – between population crosses, PL – pollen limitation, C – control, M tx Mm – crosses between *M. tommasinii* and *M. muscosa*. Dark grey cells – statistically significant p-values, light grey cells – close to significant p-values.
