# Supplementary material for: Population size as a major determinant of mating system and population genetic differentiation in a narrow endemic chasmophyte
Source: BMC Plant Biol. 2023 Aug 9;23:383. doi: 10.1186/s12870-023-04384-8 (PMC10411015; doi:10.1186/s12870-023-04384-8)
Supplement: Supplementary file 4 — Additional file 4. [file 12870_2023_4384_MOESM4_ESM.docx]

**Additional file 4**

**Table S4** Pairwise population matrix of gene flow between populations of *Moehringia muscosa* (*mus*) and *M. tommasinii* (*tom*; min: 0.33095, max: 2.1666876, Me: 0.550886).

|  |  | ***muscosa*** | | | ***tommasinii*** | | | | | |  |
| --- | --- | --- | --- | --- | --- | --- | --- | --- | --- | --- | --- |
|  |  | **Vdc^m^** | **GL^m^** | **GL** | | **CK** | **OSP** | **PP** | **ISTa** | **ISTb** | |
| ***mus*** | **Vdc^m^** | x |  |  | |  |  |  |  |  | |
|  | **GL^m^** | 1.7791002 |  |  | |  |  |  |  |  | |
| ***tom*** | **GL** | 1.2392077 | 1.2294335 |  | |  |  |  |  |  | |
|  | **CK** | 0.5492455 | 0.5575783 | 0.9941395 | |  |  |  |  |  | |
|  | **OSP** | 0.5840289 | 0.6447896 | 1.2121721 | | 2.1668764 |  |  |  |  | |
|  | **PP** | 0.5553592 | 0.6000198 | 0.5232919 | | 0.5627008 | 0.5525283 |  |  |  | |
|  | **ISTa** | 0.4527668 | 0.4529233 | 0.4364762 | | 0.3392128 | 0.3646875 | 0.3680467 |  |  | |
|  | **ISTb** | 0.4603373 | 0.4434211 | 0.4655522 | | 0.3452350 | 0.3945235 | 0.330959 | 1.7025362 |  | |

| Quartile |  |
| --- | --- |
| 1^st^ | 0.33095–0.789939 |
| 2^nd^ | 0.789939–1.24918 |
| 3^rd^ | 1.2491–1.707897 |
| 4^th^ | 1.7077899–2.1666876 |
