# Supplementary material for: Population size as a major determinant of mating system and population genetic differentiation in a narrow endemic chasmophyte
Source: BMC Plant Biol. 2023 Aug 9;23:383. doi: 10.1186/s12870-023-04384-8 (PMC10411015; doi:10.1186/s12870-023-04384-8)
Supplement: Supplementary file 5 — Additional file 5. [file 12870_2023_4384_MOESM5_ESM.docx]

**Additional file 5**

**Table S5** Bonferroni corrected p-values and Mann-Whitney pair-wise comparisons of petal index (lower left handed corner) and Nei’s genetic distances (upper right handed corner) between different populations of *M. muscosa* (populations OBR^m^, VDC^m^ and GL^m^) and *M. tommasinii* (populations GL, OSP, CK, PP, ISTa and ISTb).

|  |  | **populations** | | | | | | | | |  |
| --- | --- | --- | --- | --- | --- | --- | --- | --- | --- | --- | --- |
|  |  | ***muscosa*** | | | ***tommasinii*** | | | | | | |
|  |  | **OBR^m^** | **VDC^m^** | **GL^m^** | **GL** | **CK** | **OSP** | **PP** | **ISTa** | **ISTb** | |
| ***muscosa*** | **OBR^m^** | **x** | / | / | / | / | / | / | / | / | |
|  | **VDC^m^** | 1 | x | 0.36 | 0.57 | 1.28 | 1.11 | 1.5 | 2.22 | 1.83 | |
|  | **GL^m^** | 0.005001 | 0.009761 | x | 0.56 | 1.35 | 1.37 | 1.84 | 2.34 | 2.19 | |
| ***tommasinii*** | **GL** | 1 | 0.8486 | 1 | x | 0.47 | 0.4 | 1.66 | 1.97 | 1.51 | |
|  | **CK** | <0.0001 | <0.0001 | <0.0001 | <0.0001 | x | 0.15 | 0.89 | 2 | 1.71 | |
|  | **OSP** | <0.0001 | <0.0001 | <0.0001 | <0.0001 | 1 | x | 1.02 | 1.94 | 1.47 | |
|  | **PP** | <0.0001 | <0.0001 | <0.0001 | <0.0001 | 1 | 1 | x | 2.34 | 3.52 | |
|  | **ISTa** | <0.0001 | <0.0001 | <0.0001 | <0.0001 | 0.03855 | 0.001712 | 0.3343 | x | 0.18 | |
|  | **ISTb** | <0.0001 | <0.0001 | <0.0001 | <0.0001 | 0.04466 | 0.01473 | 0.6645 | 1 | x | |

Kruskal-Wallis test for equal medians

χ^2^: 153

χ ^2^ (tie corrected): 153

p (same): 4.719x10^-29^
