# Supplementary material for: Population size as a major determinant of mating system and population genetic differentiation in a narrow endemic chasmophyte
Source: BMC Plant Biol. 2023 Aug 9;23:383. doi: 10.1186/s12870-023-04384-8 (PMC10411015; doi:10.1186/s12870-023-04384-8)
Supplement: Supplementary file 6 — Additional file 6. [file 12870_2023_4384_MOESM6_ESM.docx]

**Additional file 6**

**Table S6** Petal length (L: mean ± SE) and width (W: mean ± SE, both in grey) in different populations of *Moehringia muscosa* and *M. tommasinii*, with Kruskal-Wallis test for equal medians and Mann-Whitney pair-wise comparisons (Bonferroni corrected p-values) for petal length (lower left handed corner) and petal width (upper right handed corner).

|  |  | **muscosa** | | | **tommasinii** | | | | | |
| --- | --- | --- | --- | --- | --- | --- | --- | --- | --- | --- |
|  |  | **OBR^m^** | **VDC^m^** | **GL^m^** | **GL** | **CK** | **OSP** | **PP** | **ISTa** | **ISTb** |
| **muscosa** | **OBR^m^** | L: 3.79±0.13  W: 1.55±0.05 | < 0.001 | < 0.001 | < 0.001 | < 0.001 | < 0.001 | < 0.001 | < 0.001 | < 0.001 |
|  | **VDC^m^** | < 0.001 | L: 4.66±0.10  W: 1.85±0.02 | < 0.001 | 1 | < 0.001 | < 0.001 | < 0.001 | < 0.001 | < 0.001 |
|  | **GL^m^** | 0.10 | 1 | L: 4.39±0.06  W: 2.13±0.04 | 0.60 | < 0.001 | < 0.001 | < 0.001 | < 0.001 | < 0.001 |
| **tomasinii** | **GL** | 0.55 | 0.53 | 1 | L: 4.27±0.12  W: 1.96±0.06 | < 0.001 | < 0.001 | < 0.001 | < 0.001 | < 0.001 |
|  | **CK** | < 0.001 | 1 | < 0.05 | < 0.05 | L: 4.92±0.11  W: 2.93±0.09 | < 0.001 | 1 | < 0.001 | < 0.001 |
|  | **OSP** | < 0.001 | < 0.001 | < 0.001 | < 0.001 | < 0.001 | L: 6.26±0.13  W: 3.85±0.09 | < 0.001 | < 0.001 | 1 |
|  | **PP** | < 0.001 | 1 | 1 | 0.92 | 1 | < 0.001 | L: 4.71±0.11  W: 3.07±0.09 | < 0.001 | < 0.001 |
|  | **ISTa** | < 0.001 | < 0.001 | < 0.001 | < 0.001 | < 0.001 | 1 | < 0.001 | L: 6.18±0.12  W: 4.36±0.05 | 0.15 |
|  | **ISTb** | < 0.001 | < 0.001 | < 0.001 | < 0.001 | 0.06 | < 0.05 | < 0.05 | < 0.05 | L: 5.56±0.13  W: 4.06±0.12 |

Kruskal-Wallis test for equal medians, petal length: χ^2^ = 128.7, p < 0.001; petal width: χ^2^ = 171.8, p < 0.001;
