# Supplementary material for: Population size as a major determinant of mating system and population genetic differentiation in a narrow endemic chasmophyte
Source: BMC Plant Biol. 2023 Aug 9;23:383. doi: 10.1186/s12870-023-04384-8 (PMC10411015; doi:10.1186/s12870-023-04384-8)
Supplement: Supplementary file 7 — Additional file 7. [file 12870_2023_4384_MOESM7_ESM.docx]

**Additional file 7**

**Table S7** Bonferroni corrected p-values and Mann-Whitney pair-wise comparisons of pollen-to-ovule ratios (P/O ratios) between different populations of *M. muscosa* (population VDC^m^) and *M. tommasinii* (populations GL, OSP, CK, PP, ISTa and ISTb).

|  | **populations** | | | | | | |
| --- | --- | --- | --- | --- | --- | --- | --- |
|  | ***muscosa*** | ***tommasinii*** | | | | | |
|  | **VDC^m^** | **GL** | **OSP** | **CK** | **PP** | **ISTa** | **ISTb** |
| **VDC^m^** | x |  |  |  |  |  |  |
| **GL** | 0.8776 | x |  |  |  |  |  |
| **OSP** | 0.5088 | 0.8053 | x |  |  |  |  |
| **CK** | 0.007657 | 0.08058 | 0.004311 | x |  |  |  |
| **PP** | 0.2144 | 0.58 | 0.6749 | 0.000504 | x |  |  |
| **ISTa** | 0.003108 | 0.01086 | 0.004052 | 0.0929 | 0.000518 | x |  |
| **ISTb** | 0.1405 | 0.3075 | 0.08448 | 0.7815 | 0.04516 | 0.07412 | x |

Kruskal-Wallis test for equal medians of pollen-to-ovule ratios in different populations of *Moehringia muscosa* and *M. tommasinii*

χ^2^: 25.8

χ^2^: (tie corrected): 25.8

p (same): 0.000242
