# Supplementary material for: Population size as a major determinant of mating system and population genetic differentiation in a narrow endemic chasmophyte
Source: BMC Plant Biol. 2023 Aug 9;23:383. doi: 10.1186/s12870-023-04384-8 (PMC10411015; doi:10.1186/s12870-023-04384-8)
Supplement: Supplementary file 8 — Additional file 8. [file 12870_2023_4384_MOESM8_ESM.docx]

**Additional file 8**

**Table S8** Summary statistics of loci selected for population-genetic analysis for *Moehringia tommasinii* and *M. muscosa*.

|  |  |  |  |  | *M. tommasinii* (*N* = 24) | | | | *M. muscosa* (*N* = 24) | | | | GeneBank Acc. no. |
| --- | --- | --- | --- | --- | --- | --- | --- | --- | --- | --- | --- | --- | --- |
| Locus name | Forward primer (5´-3´) | Reverse primer (5´-3´) | Repeat motif | Size (bp) | *N*_a_ | *H*_o_ | *H*_e_ | PIC | *N*_a_ | *H*_o_ | *H*_e_ | PIC |  |
| Moe_054 | CCGAGCCGATCAGAACAGAT | CTGGCTCCTGGGTGGATTTC | AAC | 118-178 | 15 | 0.667 | 0.908 | 0.879 | 4 | 0.167 | 0.432 | 0.374 |  |
| Moe_326 | CGCGATGGAATTTCCCTCAC | AGCAGAATTGTCAATCTCCAACC | ATC | 207-237 | 6 | 0.333 | 0.725 | 0.663 | 2 | 0.333 | 0.479 | 0.359 |  |
| Moe_108 | CAAACCAACAGGCGAACACC | GCGGTCGGATAAGGAGACG | AAG | 194-242 | 13 | 0.708 | 0.883 | 0.851 | 7 | 0.542 | 0.713 | 0.662 |  |
| Moe_494 | ACATCACCCACTACTTTCTCCAT | GTGTGAGCTATCTACCTTGTTGT | AAT | 121-211 | 19 | 0.875 | 0.946 | 0.922 | 6 | 0.583 | 0.668 | 0.610 |  |
| Moe_304 | TGCAGTAGGTATCCAAGGATCA | TCGATTTGCTTCCACCACCT | AAC | 124-160 | 7 | 0.458 | 0.776 | 0.727 | 5 | 0.625 | 0.660 | 0.591 |  |
| Moe_374 | TGGTGATGATTTAAGTGTCCCAA | TGACCCTTCCTCGTCTTCCT | AAG | 192-216 | 7 | 0.792 | 0.841 | 0.800 | 3 | 0.542 | 0.531 | 0.405 |  |
| Moe_323 | ACTGCAACTGCTTCAACTCCT | GCAAAGTGTTCAAAGTTTCGCT | AAC | 88-133 | 5 | 0.292 | 0.462 | 0.406 | 4 | 0.833 | 0.700 | 0.628 |  |
| Moe_354 | TCTCAAACTTAGACTGAGGAGGT | AACCGCCGTCATCGATTTCT | ACC | 111-123 | 4 | 0.167 | 0.556 | 0.465 | 3 | 0.125 | 0.531 | 0.405 |  |
| Moe_307 | GTGGAGCGGTATGACAAGCT | GCAATGTCGTTTACACCTAGTGA | ATC | 266-278 | 4 | 0.292 | 0.611 | 0.532 | 2 | 0.333 | 0.422 | 0.328 |  |
| Moe_246 | TGATGGGTGGAGGAATGATGA | CCAACAATGGTGCCACTCTG | ACT | 212-233 | 7 | 0.292 | 0.679 | 0.628 | 3 | 0.292 | 0.531 | 0.405 |  |

*N* – sample size, *N*_a_ – number of alleles per locus, *H*_o_ – observed heterozygosity, *H*_e_ – expected heterozygosity, PIC – polymorphic information content).

Development and characterization of new microsatellite markers for *Moehringia tommasinii* and *M. muscosa*

Since no microsatellite molecular markers were available for *Moehringia tommasinii* or any closely related species, a set of microsatellite markers was developed so that reliable assessment of the species’ population-genetic structure could be performed. After the sampling, the leaf tissue of collected individuals was immediately stored in silica gel for rapid desiccation. Genomic DNA was extracted using GenElute™ Plant Genomic DNA Miniprep Kit (Sigma-Aldrich®). DNA isolates were submitted to the AllGenetics & Biology SL (A Coruna, Spain) for the library development, sequencing, and loci characterization. For the development of the microsatellite library, an individual from the population CK was sequenced, while additional 48 samples from different populations of both studied species were used for loci characterization. Voucher specimen is deposited at the herbarium of the Natural History Museum Rijeka (NHMR 3164). A library was prepared using the Nextera XT DNA kit (Illumina), following the manufacturer's instructions. The library was enriched by hybridization with the AC, AG, ACG, and ATCT microsatellite motifs. The sequencing was performed using a 2 × 150 paired‐end protocol on an Illumina MiSeq (Illumina, San Diego, California, USA). CLC Genomics Server software (ver. 10.0.1) was used for removal of the adapter sequences and trimming, followed by the de novo assembly. QDD 3.1 software was employed for the identification of microsatellite regions within the assembled contigs. Primer pairs were developed with the Primer3 program [1] as implemented in QDD 3.1 software [2]. Default settings were used, with product size range 100-300 bp, GC content between 30–70%, and melting temperature (Tm) between 57–62°C. Only loci with pure microsatellites were considered for primer development. For initial loci characterization, PCR testing of 48 selected primer pairs was performed on five randomly selected *M. tommasinii* and *M. muscosa* individuals. Before the amplification of the entire sample set of 216 studied individuals, ten loci characterized by optimal amplification patterns and satisfying polymorphism levels were selected and additionally tested on 24 individuals from both studied species. All PCR reactions were carried out following Schuelke [3], which implies the usage of a fluorescently-labelled oligonucleotide identical to the 5' tail of the reverse primer. The oligonucleotide tails used were the universal sequences M13 (GGA AAC AGC TAT GAC CAT), CAG (CAG TCG GGC GTC ATC), and T3 (AAT TAA CCC TCA CTA AAG GG). The three oligonucleotides were labelled with the HEX dye, the FAM dye, and the TAMRA dye, respectively. Loci amplifications were carried out on the GenAmp® PCR System 9700 (Applied Biosystems, Foster City, CA, USA) using a two-step protocol with an initial touchdown cycle with following cycling conditions: 94 °C for 5 min; five cycles of 45 s at 94 °C, 30 s at 60 °C for the first cycle and 1 °C less in each subsequent cycle, and 90 s at 72 °C; 25 cycles of 45 s at 94 °C, 30 s at 55 °C, and 90 s at 72 °C; and an 8 min extension step at 72 °C. Finally, obtained PCR products were run on an ABI 3730XL (Applied Biosystems, Foster City, CA, USA) and the results were analysed and scored using GeneMapper 4.0 software (Applied Biosystems, Foster City, CA, USA).

For each microsatellite locus, basic population-genetic parameters (number of alleles per locus, the observed heterozygosity, the expected heterozygosity, and the polymorphic information content) were calculated using Cervus 3.0.7 software [4]. The obtained summary statistics of loci selected for population-genetic analysis is given in Table S7.

**Literature**

1. Rozen S, Skaletsky H: Primer3 on the WWW for General Users and for Biologist Programmers. In: *Bioinformatics Methods and Protocols.* Edited by Misener S, Krawetz SA. Totowa, NJ: Humana Press; 1999: 365-386.

2. Meglécz E, Pech N, Gilles A, Dubut V, Hingamp P, Trilles A, Grenier R, Martin J-F: QDD version 3.1: a user-friendly computer program for microsatellite selection and primer design revisited: experimental validation of variables determining genotyping success rate. *Molecular ecology resources* 2014, 14(6):1302-1313.

3. Schuelke M: An economic method for the fluorescent labeling of PCR fragments. *Nature Biotechnology* 2000, 18(2):233-234.

4. Kalinowski S, T., Taper ML, Marshall TC: Revising how the computer program cervus accommodates genotyping error increases success in paternity assignment. *Molecular Ecology* 2007, 16(5):1099-1106.
