# Supplementary material for: Population size as a major determinant of mating system and population genetic differentiation in a narrow endemic chasmophyte
Source: BMC Plant Biol. 2023 Aug 9;23:383. doi: 10.1186/s12870-023-04384-8 (PMC10411015; doi:10.1186/s12870-023-04384-8)
Supplement: Supplementary file 9 — Additional file 9. [file 12870_2023_4384_MOESM9_ESM.docx]

**Supplementary information – legends/additional legends**

**Additional file 1: Table S1:** GLM of controlled hand pollination treatments with *Moehringia tommasinii* drawn across all populations.

**Additional file 2: Table S2:** GLM of controlled hand pollination treatments with *M. tommasinii* for each population separately.

**Additional file 3: Table S3:** GLM of pollination treatments with *M. tommasinii* and *M. muscosa* – multiple comparisons of means.

**Additional file 4: Table S4:** Pairwise population matrix of gene flow between *Moehringia muscosa* and *M. tommasinii*.

**Additional file 5: Table S5**: Bonferroni corrected p-values and Mann–Whitney pair-wise comparisons of petal index and Nei’s genetic distances between *M. muscosa* and *M. tommasinii*.

**Additional file 6: Table S6:** Petal length (L: mean ± SE) and width (W: mean ± SE, both in grey) in different populations of *Moehringia muscosa* and *M. tommasinii*, with Kruskal-Wallis test for equal medians and Mann-Whitney pair-wise comparisons (Bonferroni corrected p-values) for petal length (lower left handed corner) and petal width (upper right handed corner).

**Additional file 7: Table S7**: Bonferroni corrected p–values and Mann–Whitney pair-wise comparisons of P/O ratio between populations of *M. muscosa* and *M. tommasinii*.

**Additional file 8: Table S8**: Summary statistics of loci selected for population-genetic analysis and microsatellite development.
